# Supplementary material for: The molecular architecture of cell cycle arrest
Source: Mol Syst Biol. 2022 Sep 26;18(9):e11087. doi: 10.15252/msb.202211087 (PMC9511499; doi:10.15252/msb.202211087)
Supplement: Supplementary file 1 — Expanded View Figures PDF [file MSB-18-e11087-s004.pdf]

Expanded View Figures

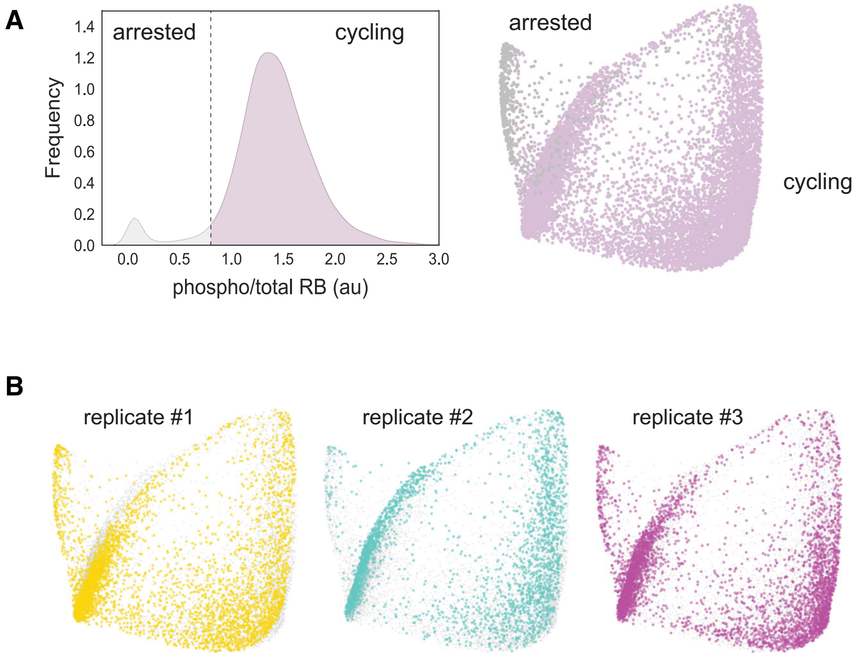

Figure EV1. The cell cycle map of unperturbed RPE cells.

A *Left*: Distribution of nuclear intensity ratios of phospho/total RB in unperturbed RPE cells. A threshold value of 0.7 was used to label cells as arrested (low phospho/total RB) or actively cycling (high phospho/total RB). *Right*: Cycling and arrested labels are overlaid on the cell cycle map.

B Cells from three technical replicates are labeled on the cell cycle map.

Figure EV2. Arrest trajectories following replication stress.

A Cell cycle map arrest of unperturbed (gray) and etoposide-treated cells (1 μM; 1 day: light green, 2 days: green, 3 days: dark green, 4 days: darker green). The unperturbed cell cycle (dotted gray line) and two arrest trajectories (into 2C and 4C, pink and purple, respectively) are indicated on the map.

B–E (B) Phospho-H2AX, (C) phospho-CHK1, (D) phospho-p65 and (E) p53 of unperturbed (left panels) or etoposide-treated cells (right panels) are plotted on the arrest architecture. Median nuclear values are shown.

F, G Heatmap of feature intensity along the (F) 2C and (G) 4C arrest trajectories. Features were ordered by hierarchical clustering according to their dynamics along each arrest trajectory. Diffusion pseudotime values were binned and pseudotime values with < 15 cells were excluded from the visualization.

H *Top*: Nuclear phospho-RB intensities of control and etoposide-treated MCF10A cells (7 days, 1 μM). Dashed-red box indicates arrested etoposide-treated cells (phospho-RB < 500 au). *Bottom*: Distribution of DNA content in arrested etoposide-treated cells. Arrested MCF10A cells were observed with both 2C and 4C DNA content.

© 2022 The Authors

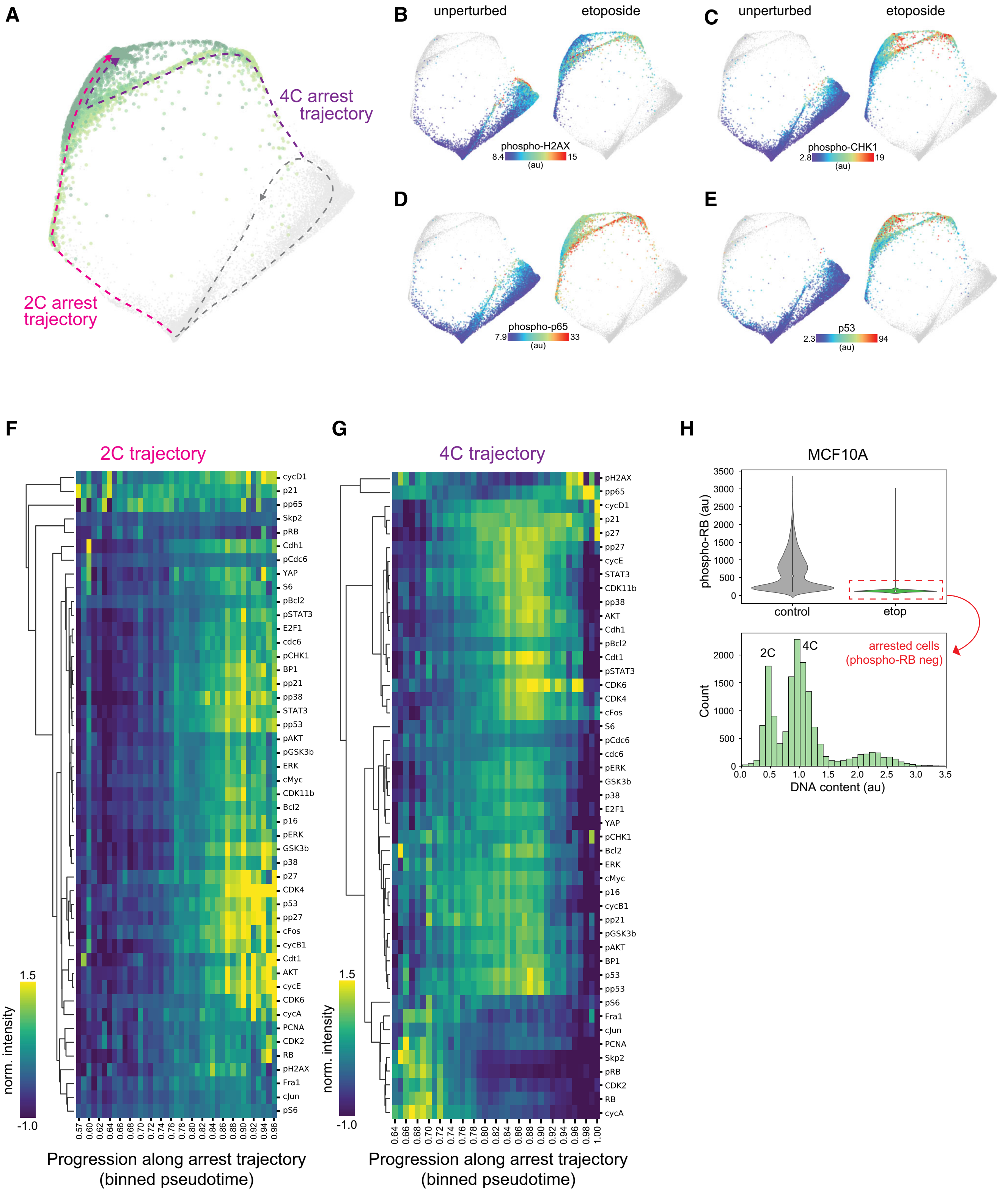

Figure EV2.

©2022 The Authors

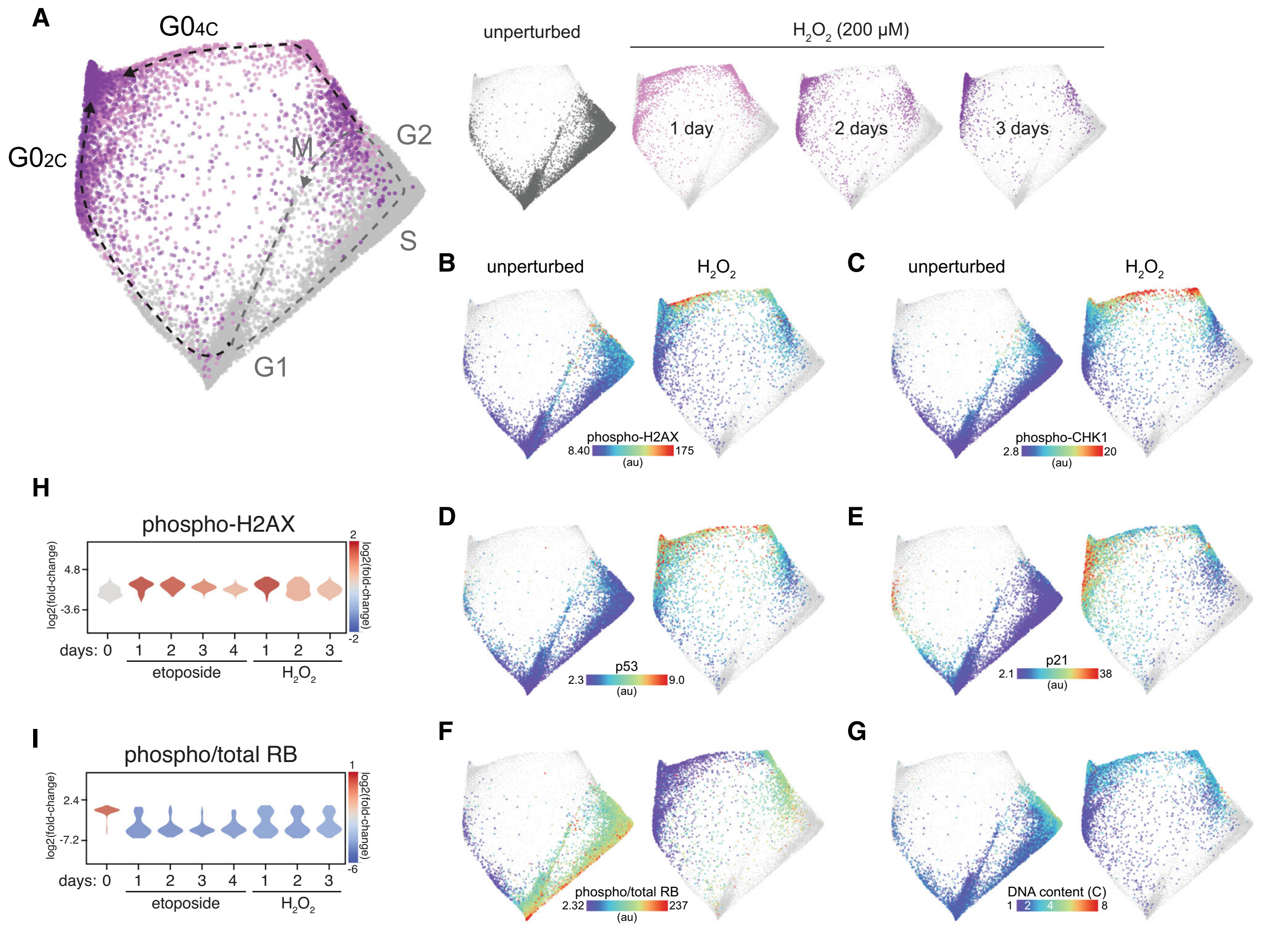

Figure EV3. The arrest architecture of oxidative stress.

A Unified cell cycle map arrest of unperturbed (gray) and H_2O_2-treated cells (200 μM; 1 day: light purple, 2 days: purple, 3 days: dark purple – see inset, *N* = 5,015 cells). The unperturbed cell cycle (dotted gray line) and two arrest trajectories (into $G0_{2C}$ and $G0_{4C}$; black dotted lines) are indicated on the map. Inset: Each condition is shown individually on the map (other conditions are shown in lighter gray).

B–G (B) Phospho-H2AX, (C) phospho-CHK1, (D) p53, (E) p21, (F) phospho/total RB and (G) DNA content of unperturbed (left panels) or H_2O_2-treated cells (right panels) are plotted on the arrest architecture. Median nuclear values are shown for B-F.

H, I Distribution of (H) phospho-H2AX and (I) phospho/total RB in individual cells following etoposide (1 μM) or H_2O_2 treatment (200 μM).

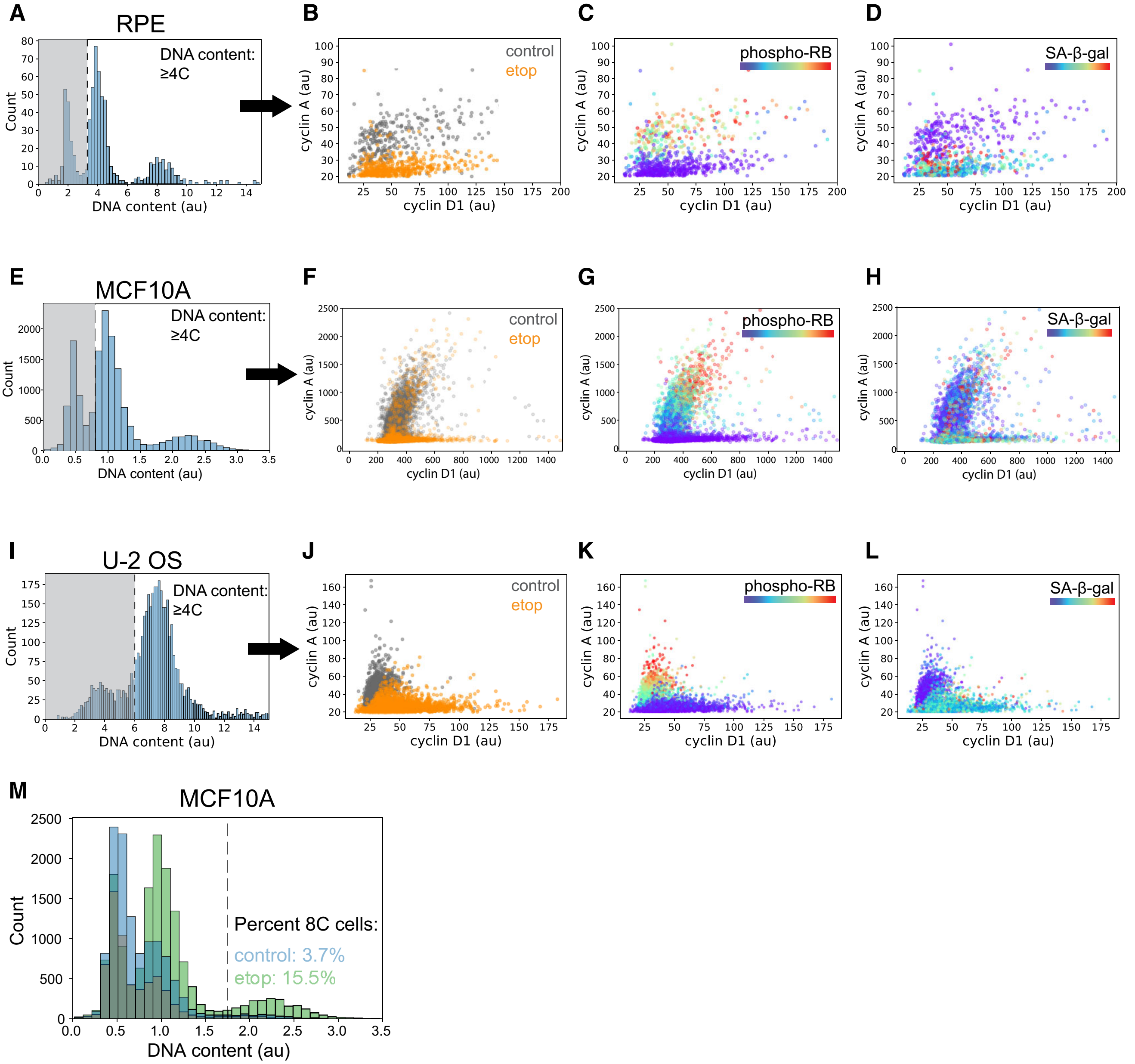

Figure EV4. Replication stress induces mitotic skipping in RPE, MCF10A and U-2 OS cells.

A–L (A) RPE cells with ≥4C DNA content were selected using the intensity of nuclear Hoechst staining. Cyclin A versus cyclin D1 intensity was plotted for each cell and overlaid with (B) condition labels (control vs. etoposide), (C) phospho-RB intensity and (D) senescence-associated β-galactosidase (SA-βgal) activity. The same analysis as above was performed on (E–H) MCF10A and (I–L) U-2 OS cells.

M Distribution of DNA content in control (blue) and etoposide-treated (green, 7 days, 1 μM) MCF10A cells. The percent of polyploid cells with DNA content = 8C is shown for both conditions.

 ©2022 The Authors

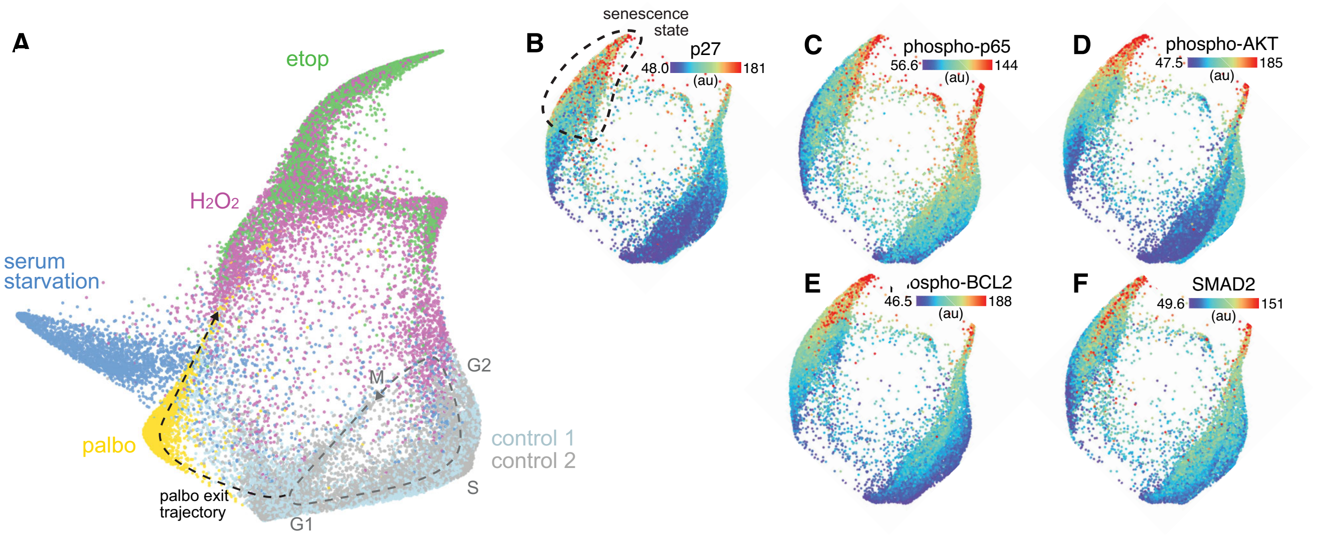

Figure EV5. The arrest architecture of palbociclib-induced arrest.

A Unified cell cycle map of unperturbed (control 2, gray) and palbociclib-treated cells (gold) from a separate experiment, plotted with unperturbed (control 1: light blue, from original experiment), serum-starved (blue) etoposide- (etop, green) and H_2O_2-treated (magenta) cells from initial experiment. Data integration is described in Materials and Methods.

B–F (B) p27, (C) phospho-p65, (D) phospho-AKT, (E) phospho-BCL2 and (F) SMAD2 are plotted on the map. Median nuclear values are shown. Dotted area indicates senescent region.

© 2022 The Authors
