# Supplementary material for: The molecular architecture of cell cycle arrest
Source: Mol Syst Biol. 2022 Sep 26;18(9):e11087. doi: 10.15252/msb.202211087 (PMC9511499; doi:10.15252/msb.202211087)
Supplement: Supplementary file 4 — PDF+ [file MSB-18-e11087-s003.pdf]

Article

TRANSPARENT PROCESS
OPEN ACCESS

molecular systems biology

The molecular architecture of cell cycle arrest

Wayne Stallaert[1,2,†], Sovanny R Taylor[1,2], Katarzyna M Kedziora[1,3], Colin D Taylor[1,2], Holly K Sobon[1,2], Catherine L Young[1,2], Juanita C Limas[4], Jonah Varblow Holloway[1,2], Martha S Johnson[4], Jeanette Gowen Cook[4,5] & Jeremy E Purvis[1,2,*]

Abstract

The cellular decision governing the transition between proliferative and arrested states is crucial to the development and function of every tissue. While the molecular mechanisms that regulate the proliferative cell cycle are well established, we know comparatively little about what happens to cells as they diverge into cell cycle arrest. We performed hyperplexed imaging of 47 cell cycle effectors to obtain a map of the molecular architecture that governs cell cycle exit and progression into reversible ("quiescent") and irreversible ("senescent") arrest states. Using this map, we found multiple points of divergence from the proliferative cell cycle; identified stress-specific states of arrest; and resolved the molecular mechanisms governing these fate decisions, which we validated by single-cell, time-lapse imaging. Notably, we found that cells can exit into senescence from either G1 or G2; however, both subpopulations converge onto a single senescent state with a G1-like molecular signature. Cells can escape from this "irreversible" arrest state through the upregulation of G1 cyclins. This map provides a more comprehensive understanding of the overall organization of cell proliferation and arrest.

Keywords cell cycle; proliferation; quiescence; senescence; single-cell
Subject Category Cell Cycle
DOI 10.15252/msb.202211087 | Received 22 April 2022 | Revised 7 September 2022 | Accepted 9 September 2022
Mol Syst Biol. (2022) 18: e11087

Introduction

The decision of when and where to trigger cell division is fundamental to nearly all aspects of development and physiology. At the level of the individual cell, the molecular basis of the proliferation/arrest decision is embedded within a highly interconnected and dynamic network of cell cycle regulators. Progression through the proliferative phases of the cell cycle (G1/S/G2/M) is governed by a series of biochemical reactions that are coordinated in time and space to ensure the successful replication of DNA and its division into two daughter cells. In addition to these four proliferative phases, cells may also "exit" the proliferative cell cycle into a state of cell cycle arrest, often referred to as G0. While arrested, cells still perform many essential cellular functions including metabolism, secretion, transcription, and translation. However, as long as they remain in the G0 state, arrested cells neither synthesize DNA nor undergo cell division. This five-state model has become the canonical cell cycle model found in most textbooks (Morgan, 2007) and the current literature (Spencer *et al*, 2013; Overton *et al*, 2014; Marescal & Cheeseman, 2020) and has shaped our thinking about the cell cycle for over 70 years (Howard & Pelc, 1951; Cameron & Greulich, 1963; Smith & Martin, 1973).

While the mechanisms that govern progression through the proliferative cell cycle have been studied extensively, we know comparatively little about what happens to cells after they exit the proliferative cell cycle. We know that cells may exit the cell cycle in response to various biochemical (e.g., DNA damage and oxidative stress) or environmental insults (e.g., lack of mitogens and high local cell density) triggered by different molecular mechanisms (Sagot & Laporte, 2019; Marescal & Cheeseman, 2020). After exiting the cell cycle, cells may progress into deeper states of reversible ("quiescent") cell cycle arrest (Owen *et al*, 1989; Kwon *et al*, 2017; Wang *et al*, 2017), and in some cases can transition into an irreversible ("senescent") state of arrest (Marthandan *et al*, 2014; Sousa-Victor *et al*, 2014; Fujimaki *et al*, 2019; Fujimaki & Yao, 2020). Clearly, cell cycle arrest is far from a single, static molecular state (Coller *et al*, 2006; Klosinska *et al*, 2011; Sun & Buttitta, 2017), yet a systematic characterization of when and how cells arrest remains lacking.

In this study, we used a combination of hyperplexed, single-cell imaging and manifold learning to map the molecular architecture of cell cycle arrest. Previously, we used this approach to map the structure of the proliferative cell cycle in unperturbed, nontransformed retinal pigment epithelial (RPE) cells (Stallaert *et al*, 2022). Building upon this work, here, we exposed asynchronous RPE cells to three distinct stressors—hypomitogenic, replication, and oxidative—known to induce cell cycle arrest. For each stress, we identify the

1 Department of Genetics, University of North Carolina at Chapel Hill, Chapel Hill, NC, USA
2 Computational Medicine Program, University of North Carolina at Chapel Hill, Chapel Hill, NC, USA
3 Bioinformatics and Analytics Research Collaborative (BARC), University of North Carolina at Chapel Hill, Chapel Hill, NC, USA
4 Department of Biochemistry and Biophysics, University of North Carolina at Chapel Hill, Chapel Hill, NC, USA
5 Department of Pharmacology, University of North Carolina at Chapel Hill, Chapel Hill, NC, USA
*Corresponding author. Tel: +919 962 4923; E-mail: jeremy_purvis@med.unc.edu
†Present address: Department of Computational and Systems Biology, University of Pittsburgh, Pittsburgh, PA, USA

©2022 The Authors. Published under the terms of the CC BY 4.0 license.

points of exit from the proliferative cell cycle, the mechanism(s) that induced arrest, and the molecular signatures of cells as they transition through distinct arrest states. We reveal a complex architecture of molecular trajectories through arrest state space and identify states of arrest not observed in our previous mapping of the human cell cycle. We show that cells exit the cell cycle along two distinct arrest trajectories in response to replicative and oxidative stress and that these trajectories are distinct from the arrest state induced by hypomitogenic stress. We demonstrate how sustained replication stress can generate polyploid cells through mitotic skipping and endoreduplication. Finally, we identify the molecular trajectories that lead to "irreversible" arrest and reveal that cellular senescence is an obligate G1-like molecular state that can be reversed by increasing the expression of G1 cyclins.

Results

To map the molecular architecture of cell cycle arrest, we subjected an asynchronous population of RPE cells to a variety of natural stresses known to induce exit from the proliferative cell cycle. These stresses included hypomitogenic stress (induced by serum starvation), replication stress (using the topoisomerase inhibitor etoposide), and oxidative stress (by exogenous H_2O_2 addition). We performed iterative indirect immunofluorescence imaging (4i) (Gut *et al*, 2018) of 47 cell cycle effectors (Table EV1) and DNA. From these 48 images, we extracted 2,952 unique single-cell features, including the subcellular expression of each protein across different cellular compartments (i.e., nucleus, cytosol, plasma membrane, and perinuclear region) as well as cell morphological features, such as size and shape, for 23,605 individual cells (Fig 1A). After feature selection (to identify features that vary in a cell-cycle-dependent manner; Stallaert *et al*, 2022), we performed manifold learning using Potential of Heat-diffusion for Affinity-based Transition Embedding (PHATE; Moon *et al*, 2019). Manifold learning techniques such as PHATE are used to find the "surface" within this high-dimensional feature space that represents progression through the cell cycle. In other words, by placing cells with similar cell cycle signatures close to one another in a lower-dimensional (2-d) space, we can piece together the paths they take through the cell cycle and the molecular changes that accompany them. In this manuscript, we will use these lower-dimensional embeddings or cell cycle "maps," to identify the points at which cells exit the proliferative cell cycle in response to each stress and the mechanisms governing these proliferation/arrest decisions.

To identify the precise molecular states in which proliferating cells exit the cell cycle, we first resolved the unperturbed cell cycle as a reference map (Fig 1B–I). For each cell, phase annotations were obtained for the proliferative cell cycle (G1/S/G2/M; Fig 1B) using a Gaussian mixture model trained on cell cycle features previously shown to vary by phase (Stallaert *et al*, 2022). Arrested G0 cells were identified by thresholding on the phosphorylated fraction of RB (Figs 1B and EV1A), which distinguishes arrested from actively cycling cells. Using diffusion pseudotime, a nonlinear trajectory inference method that orders individual cells by their position in high-dimensional feature space and can resolve branching points in these trajectories (Haghverdi *et al*, 2016), we observed two principal trajectories: a cyclical proliferative trajectory and a single trajectory into cell cycle arrest (Fig 1C). This overall structure was reproducible across experimental replicates (Fig EV1B) and is consistent with an emerging model of the cell cycle in which cells bifurcate along two distinct trajectories following cell division (Spencer *et al*, 2013; Yang *et al*, 2017; Stallaert *et al*, 2022). Some cells maintain high RB phosphorylation (Fig 1D) and immediately reenter the proliferative cell cycle, through which we observed a doubling of DNA content (Fig 1E) and characteristic dynamics in cell cycle effectors, including cyclins D1, A and B1 (Fig 1F–H). Other cells diverge from the proliferative trajectory soon after cell division into a state of arrest that is accompanied by an abrupt dephosphorylation of RB (Fig 1D) and an increase in p21 (Fig 1I). This is the only state of arrest that we observed in unperturbed cells. Previous studies have shown that this "spontaneous" cell cycle arrest is driven by low levels of endogenous stress (including replication stress) during the mother cell cycle (Arora *et al*, 2017; Min & Spencer, 2019).

Hypomitogenic stress

To induce hypomitogenic stress, cells were serum-starved for 1 or 7 days prior to fixation. To show how hypomitogenic stress disrupts the normal cycling of cells, we generated a new cell cycle map by performing manifold learning on the combined data from unperturbed (Fig 2A, dark gray) and serum-starved cells (Fig 2A, light and dark blue). This new embedding effectively "repositions" the unperturbed cell cycle (from Fig 1) relative to a new and distinct state of cell cycle arrest ("hypomitogenic G0") that appears only in response to serum starvation. Trajectory inference by diffusion pseudotime revealed that serum-starved cells diverge from the proliferative cell cycle during G2 (Fig 2B). Unlike spontaneous arrest, this cell cycle exit was not accompanied by a large increase in p21 (Fig 2C). In the unperturbed cell cycle, cyclin D1 increased in late G2 and remained elevated during mitosis and after cell division (Fig 2D, left panel), as previously observed (Gookin *et al*, 2017; Stallaert *et al*, 2022). After serum starvation, cyclin D1 remained comparatively low during G2 (Fig 2D, right panel) and cells underwent mitosis (as indicated by a drop in DNA content; Fig 2E) directly into a state of arrest with low RB phosphorylation (Fig 2F). To validate this mechanism of cell cycle exit in individual living cells, we performed time-lapse imaging of RPE cells expressing cyclin D1 tagged with a fluorophore at its endogenous locus (cycD1-Venus), as well as a fluorescent biosensor of CDK2 activity (DHB-mCherry), which can be used to distinguish actively proliferative versus arrested cells (Spencer *et al*, 2013). While unperturbed cells exhibited a clear increase in cyclin D1 during G2, serum starvation significantly reduced the induction of cyclin D1 during G2 and in daughter cells following cell division (Fig 2G). This decrease in cyclin D1 protein in daughter cells following serum starvation was previously shown to result from a decrease in cyclin D1 mRNA during the mother cell cycle (Guo *et al*, 2005; Yang *et al*, 2017).

After exiting the cell cycle, progression further along the hypomitogenic arrest trajectory was accompanied by a decrease in the abundance of nearly every protein measured, including key proliferative effectors such as CDK2, CDK4, CDK6, CDH1, CDT1, PCNA, SKP2, FRA1, and cJUN, as well as decreased nuclear YAP and mTOR signaling (S6 phosphorylation) (Fig 2H). The only proteins

 ©2022 The Authors

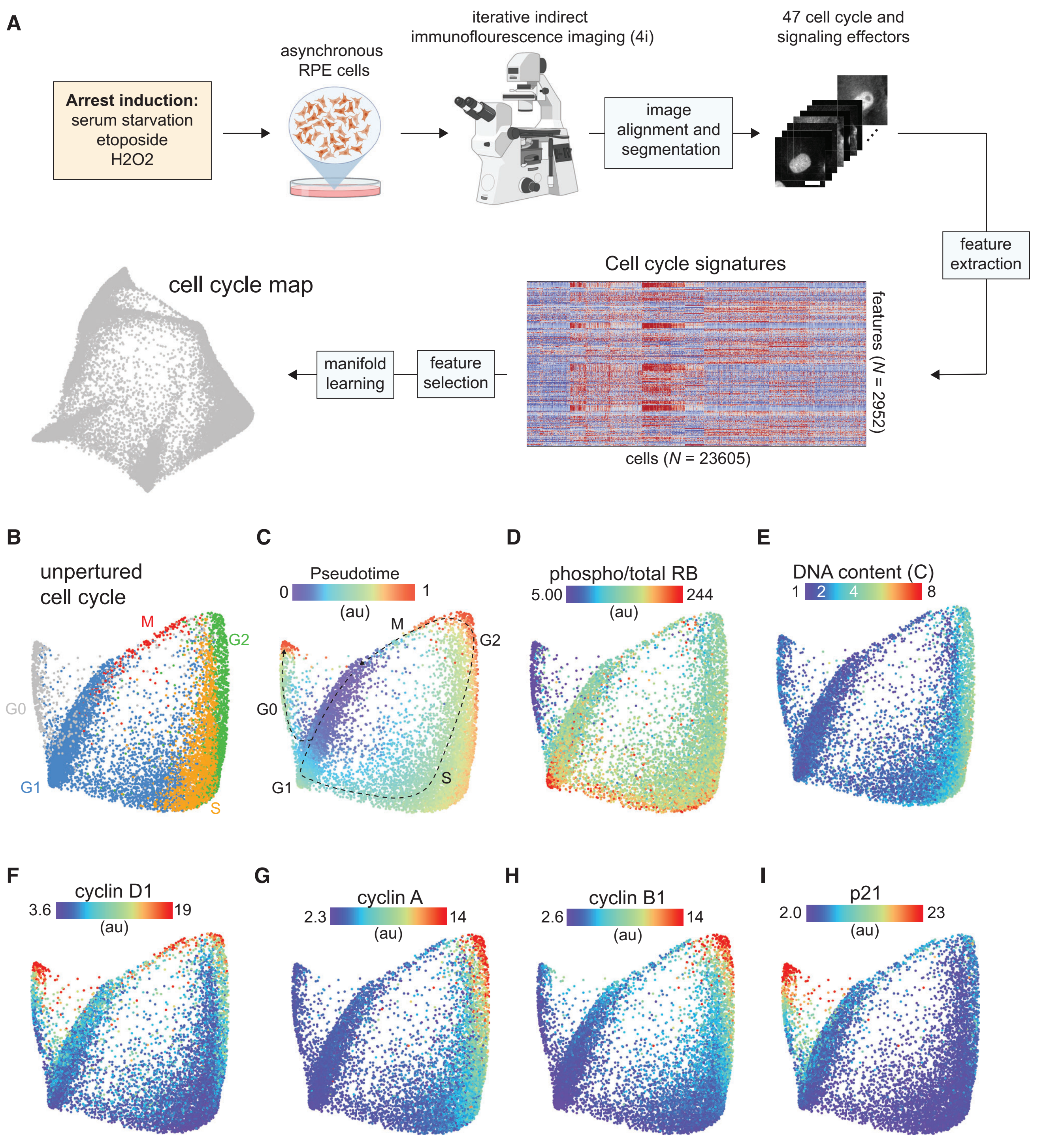

Figure 1. Mapping the architecture of cell cycle arrest.

A Schematic of the experimental approach.

B Cell cycle map of unperturbed cells (*N* = 11,268 cells). Proliferative (G1/S/G2/M) and arrested (G0) cell cycle phases were predicted for each cell using a Gaussian-mixture model and labeled on the map.

C–I (C) Diffusion pseudotime values, (D) phospho/total RB, (E) DNA content, (F) cyclin D1, (G) cyclin A, (H) cyclin B1 and (I) p21 of unperturbed cells are plotted on the map. Median nuclear values are shown for (D–I).

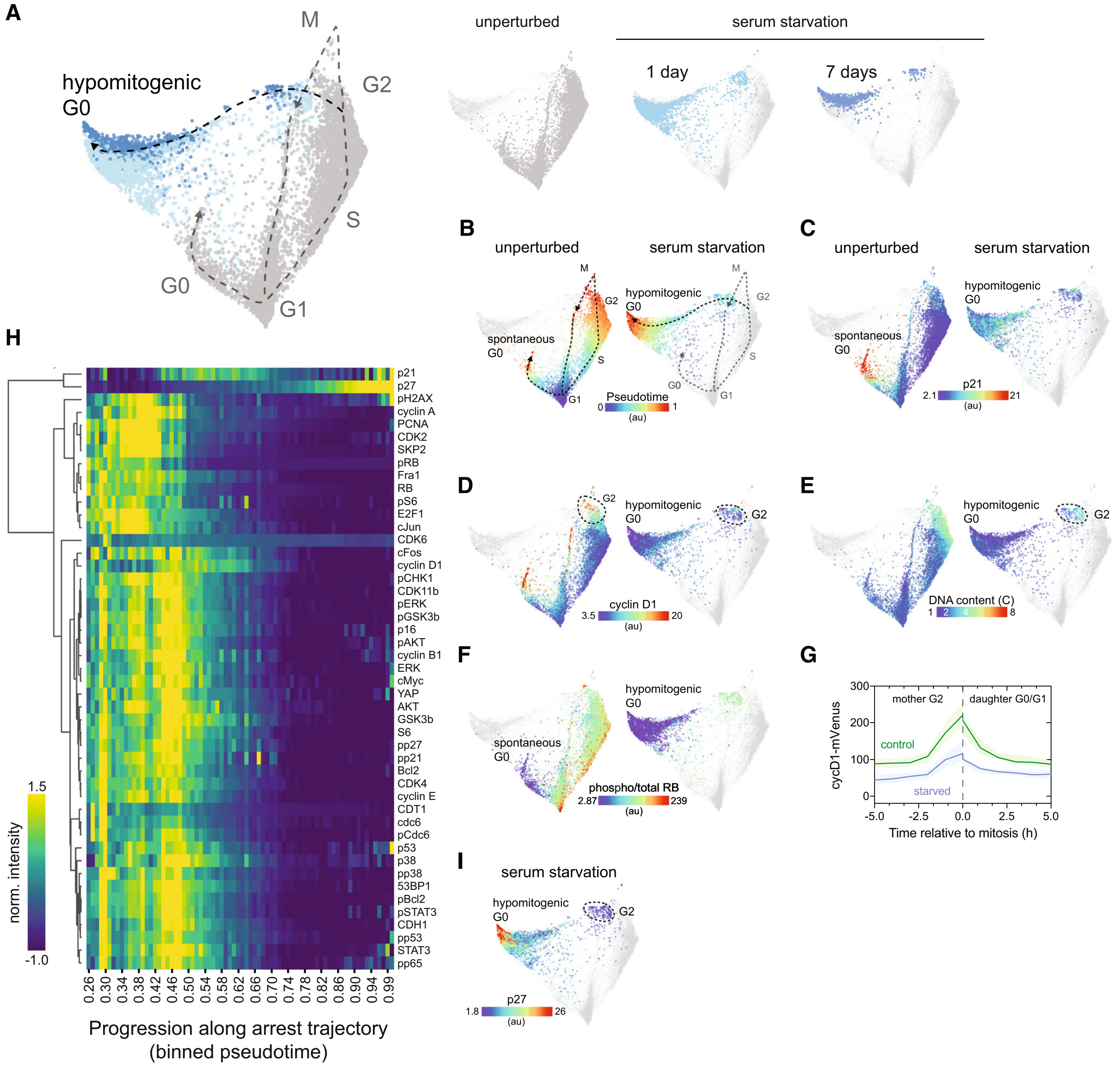

Figure 2. The arrest architecture of hypomitogenic stress.

A Unified cell cycle map of unperturbed (gray) and serum-starved cells (1 day: light blue, 7 days: dark blue, N = 3,007 cells). The proliferative cell cycle (dotted gray line) and the hypomitogenic arrest trajectory (black dotted line) are indicated on the map. Inset: Each treatment condition is shown individually on the unified map (other conditions are shown in lighter gray).

B–F (B) Diffusion pseudotime, (C) p21, (D) cyclin D1, (E) DNA content and (F) phospho/total RB of unperturbed (left panels) or serum-starved cells (right panels) are plotted on the map.

G Time-lapse imaging of cyclinD1-mVenus intensity in unperturbed (control, green) and serum-starved cells (blue). Cells were serum-starved for at least 8 h prior to imaging. The solid line represents the population median and the shaded area indicates the 95% confidence interval. N = 105 control cells and N = 111 starved cells.

H Heatmap of feature intensity along the hypomitogenic arrest trajectory. Features were ordered by hierarchical clustering according to their dynamics along the arrest trajectory. Diffusion pseudotime values were binned and pseudotime values with < 15 cells were excluded from the visualization.

I Median nuclear p27 abundance in serum-starved cells is plotted on the map.

not downregulated following serum starvation were the CDK inhibitor proteins p27 (Fig 2H–I) and, to a much lesser extent, p21 (Fig 2C and H). In fact, the abundance of p27 gradually increased as cells progressed further along the arrest trajectory, consistent with a previous study showing an increase in p27 in murine fibroblasts following serum starvation (Coats *et al*, 1996).

 ©2022 The Authors

Replication stress

To induce replication stress, cells were treated with etoposide (1 μM), an inhibitor of DNA topoisomerase II that interferes with DNA religation step during replication, for 1–4 days prior to fixation. Once again, we constructed a new cell cycle map by performing manifold learning on the combined data from unperturbed (Fig 3A, dark gray) and etoposide-treated cells (Fig 3A, green) to show how replicative stress interferes with cell cycle progression. Within a single population of cells treated with etoposide, individual cells diverged from the proliferative cell cycle along two distinct arrest trajectories. One subpopulation exited from G2 after DNA replication was complete (DNA content = 4C). A second subpopulation exited the cell cycle in the subsequent G1 phase of daughter cells immediately following mitosis (DNA content = 2C) (Fig 3B and C). Both subpopulations entered arrest states characterized by a loss of RB phosphorylation (Fig 3D). Cell cycle exit along the 4C trajectory was accompanied by activation of the DNA damage checkpoint in G2 as indicated by an increase in markers of DNA damage signaling, including phospho-H2AX, phospho-CHK1, phospho-p65, p53 (Fig EV2A–E), and p21 (Fig 3E). By contrast, daughter cells that exited the cell cycle following mitosis along the 2C trajectory did not

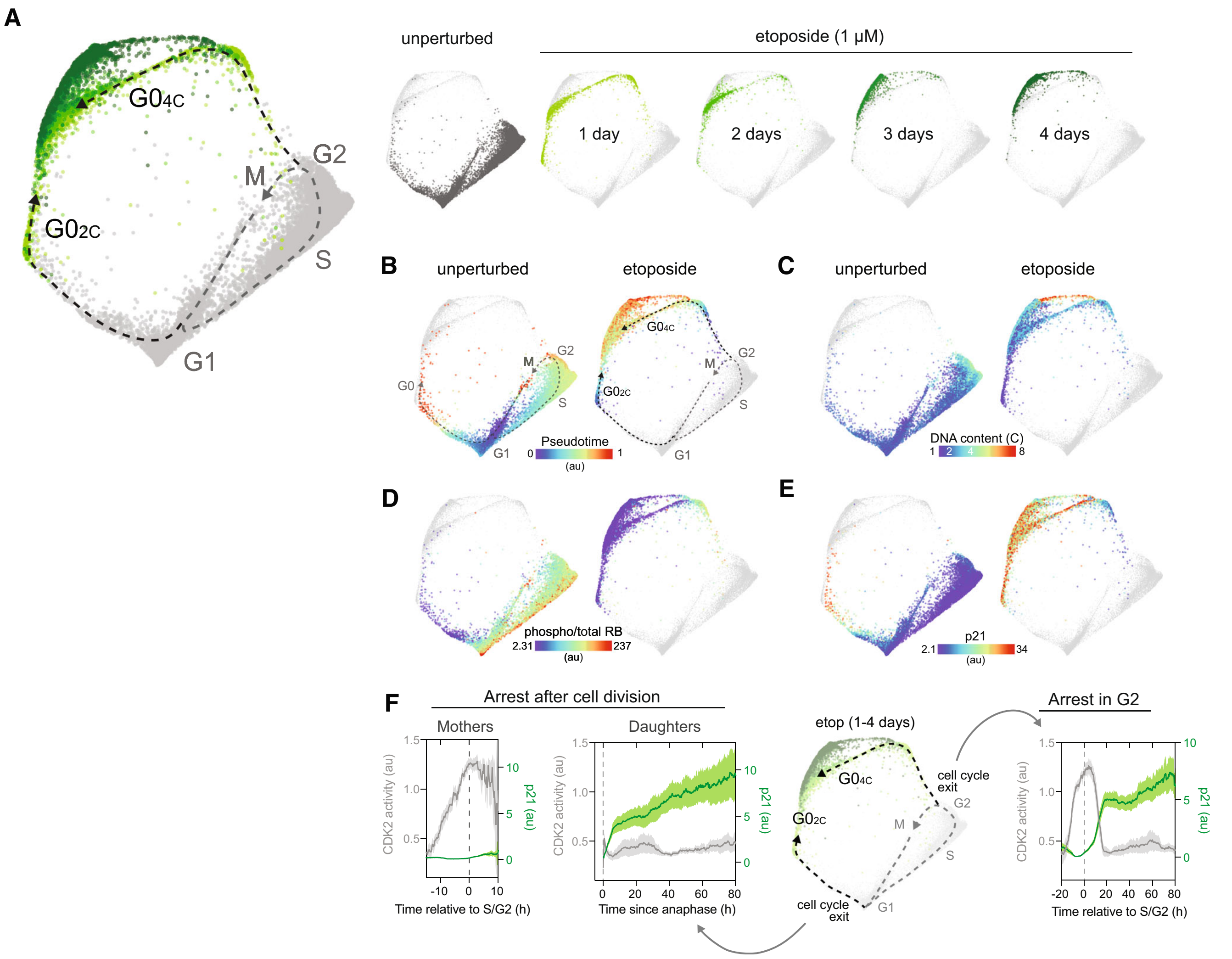

Figure 3. The arrest architecture of replication stress.

A Unified cell cycle map of unperturbed (gray) and etoposide-treated cells (1 μM; 1 day: light green, 2 days: green, 3 days: dark green, 4 days: darker green – see inset, N = 4,315 cells). The unperturbed cell cycle trajectory (dotted gray line) and two arrest trajectories (into $G0_{2C}$ and $G0_{4C}$; black dotted lines) are indicated on the map. Inset: Each condition is shown individually on the map (other conditions are shown in lighter gray).

B–E (B) Diffusion pseudotime, (C) DNA content, (D) phospho/total RB and (E) p21 of unperturbed (left panels) or etoposide-treated cells (right panels) are plotted on the arrest architecture. Median nuclear values are shown.

F Time-lapse imaging of CDK2 activity (DHB-mCherry, gray) and p21-YPet (green) intensity in etoposide-treated cells. Schematic shows the two arrest trajectories observed following etoposide treatment. Cells that successfully complete G2 ("Mothers," N = 32 cells) but arrest following cell division ("Daughters," N = 45 cells) are shown in the two leftmost panels. Cells that arrest in G2 (N = 40 cells) are shown in the rightmost panel. The solid lines represent population medians and the shaded area indicates the 95% confidence interval.

©2022 The Authors

express early markers of DNA damage signaling (phospho-H2AX, phospho-CHK1) (Fig EV2B and C), but possessed sustained elevation of phospho-p65, p53 (Fig EV2D and E) and p21 (Fig 3E), consistent with replication stress inherited from the previous cell cycle (Arora *et al*, 2017). We also observed two states of cell cycle arrest, corresponding to cell cycle exit from G1 (DNA content = 2C) or G2 (DNA content = 4C) in breast epithelial cells (MCF10A) following sustained replication stress (Fig EV2H).

To validate the observation that individual cells exit the cell cycle along two distinct arrest trajectories in response to replication stress and to investigate the mechanisms that govern this decision, we performed single-cell time-lapse imaging of RPE cells expressing a cell cycle sensor (PCNA-mTq2), a CDK2 activity sensor to detect cell cycle arrest (DHB-mCherry), and endogenous p21 fused to a fluorophore (p21-YPet) for 4 days after etoposide treatment. We observed a similar bifurcation of cell fate in live cells in response to replication stress, with 56% of cells exiting the cell cycle in G2 (along the 4C trajectory) and 44% proceeding through to mitosis following etoposide treatment (Fig 3F). The loss of CDK2 activity that accompanied cell cycle arrest in G2 occurred simultaneously with an increase in p21 expression (Fig 3F, last panel). For each of the cells that successfully progressed through to mitosis, however, no detectable p21 induction was observed in G2 (Fig 3F, first panel). Instead, their daughter cells arrested immediately following cell division (as indicated by a sustained decrease in CDK2 activity) accompanied by an increase in p21 expression shortly after cell division (Fig 3F, second panel).

Over several days of etoposide treatment cells proceed further along the 2C and 4C arrest trajectories, transitioning into additional molecular states in the upper portions of the map (Fig 3A inset), which we will discuss in greater detail below.

Oxidative stress

To assess how the cell cycle responds to oxidative stress, cells were treated with hydrogen peroxide (H_2O_2) for 1, 2 or 3 days. Overall, the arrest architecture of oxidative stress was very similar to that induced by replication stress (Fig EV3A), suggesting that the dominant cell cycle response to exogenous oxidative stress is primarily related to its ability to induce DNA damage (Demple & Halbrook, 1983). Consistent with this notion, oxidative stress induced elevated markers of the DNA damage response including phospho-H2AX, phospho-CHK1, p53 and p21 (Fig EV3B–E). Similar to etoposide treatment, H_2O_2-induced cell cycle exit along two distinct trajectories diverging from either G1 or G2, into arrest states (Fig EV3F) with 2C or 4C DNA content (Fig EV3G), respectively, both accompanied by p21 induction (Fig EV3E). However, unlike etoposide, H_2O_2 is rapidly metabolized following addition to cells (Sobotta *et al*, 2013), and thus, the resultant DNA damage is more transient. As a result, markers of DNA damage decreased more rapidly after treatment (Fig EV3H), and a higher proportion of cells remained in the cell cycle over time (Fig EV3I) compared with the sustained replication stress induced by etoposide.

Senescence, mitotic skipping, and polyploidy

Over the 4 days of etoposide treatment, cells transitioned through different arrest states (Fig 4A and B) accompanied by changes in their molecular signatures (Fig EV2F and G). In the first 1–2 days, cells populated two distinct arrest trajectories ("2C state" and "4C state") (Fig 4B). After 3–4 days, however, most cells transitioned further along these trajectories and eventually converged on a single region of the structure (see black area outlined by dotted line in Fig 4A), while a small proportion of cells also began to populate a region consisting entirely of polyploid cells ("8C state"). Similarly, after ~ 3–4 days of etoposide treatment, most cells also begin to possess elevated senescence-associated β-galactosidase (SA-β-gal) activity (Fig 4C), a hallmark of senescence (Hjelmeland *et al*, 1999). We therefore hypothesized that these regions of the map may represent irreversibly arrested senescent states. We previously identified a multivariate, proteomic signature of senescence in RPE cells (Stallaert *et al*, 2022). We used this signature to identify senescent cells following etoposide treatment. Cells in the upper-left region possessed many senescent markers including GSK3β, phospho (Thr157)- and total p27, CDK4, cyclin D1, and cyclin E (Fig 4D–I). This subpopulation contained the largest cells in the population (Fig 4J) and possessed the lowest DNA:cytoplasm ratio (Fig 4K), both hallmarks of senescent cells (Neurohr *et al*, 2019). Cells in the 8C state expressed some of these senescent features but were notably lacking p27 and CDK4, suggesting that these arrested polyploid cells reside in a different molecular state than the senescent cells in the upper-left region (Fig 4A).

We next investigated how cells that exited the cell cycle from G2 (with four copies of DNA) converged on a molecular state expressing the high levels of G1 cyclins and CDKs described above. As cells progressed along the G2 arrest trajectory, there was an abrupt degradation of the G2/M cyclins A and B (Fig 4L and M) that coincided with the loss of RB phosphorylation (Fig 3D). Degradation of G2/M cyclins normally occurs during mitosis (Glotzer *et al*, 1991); however, we observed no change in DNA content (Fig 3C) nor any visual evidence of mitotic events in the images of cells along this trajectory. Progression along the G2 trajectory was also accompanied by an increase in APC/C subunit CDH1 and a loss of SKP2 (Fig 4N and O). These molecular events are consistent with a transition into a G1-like molecular state through a phenomenon known as "mitotic skipping" (Fig 4A), which can precede senescence (Suzuki *et al*, 2012; Johmura *et al*, 2014). This phenomenon did not appear to be specific to RPE cells, as we observed that etoposide treatment induced a G1-like senescent state (high cyclin D1, low cyclin A and four copies of DNA) both in MCF10A and osteosarcoma (U-2 OS) cells as well (Fig EV4).

Next, we asked how the molecular signature of this G1-like senescent state compares with a *bone fide* G1 senescent state. Acute CDK4/6 inhibition with palbociclib (1 μM, 24 h) triggered cell cycle exit from G1 into a state of arrest distinct from hypomitogenic, replication, or oxidative stress (Fig EV5A). Sustained palbociclib treatment (4–8 days) induced senescence as measured by an increase in SA-β-gal activity (Fig 5A). To compare the G1- and G1-like senescent states induced by palbociclib and etoposide, respectively, we performed a targeted 4i experiment to generate a map of the paths that cells take into senescence in response to these specific perturbations (Fig 5B). We obtained single-cell measurements of 37 proteins, including cell cycle and signaling effectors (including cell cycle features previously shown to be upregulated in senescent cells; Stallaert *et al*, 2022), as well as several measures of the senescence-associated secretory phenotype (SASP). Again, we observed that

 ©2022 The Authors

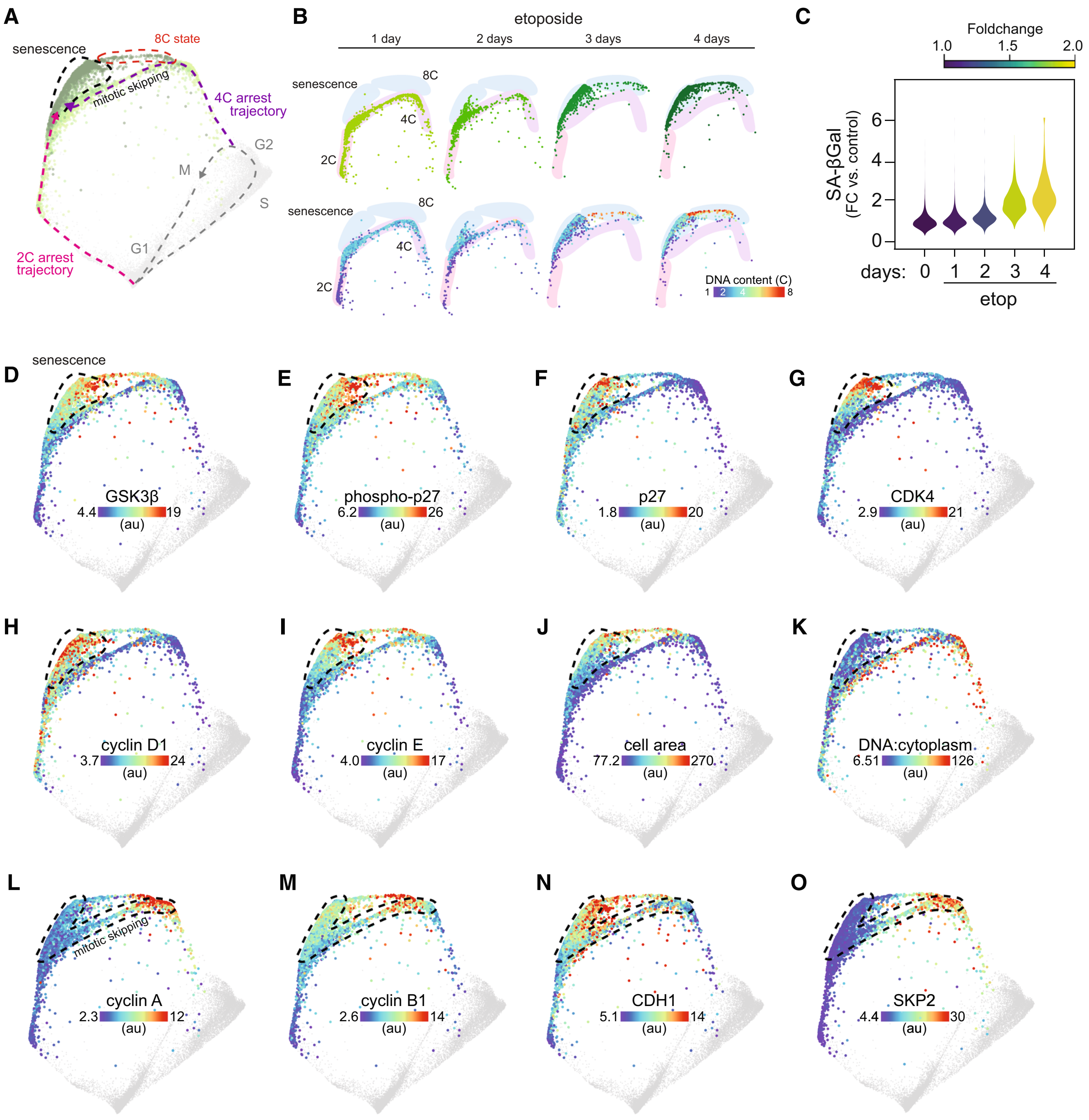

Figure 4. Replication stress promotes mitotic skipping and cellular senescence.

A Arrest architecture of replicative stress. 2C and 4C arrest trajectories are shown, the regions that correspond to mitotic skipping and the senescent state are annotated and the arrest trajectory of 8C polyploid cells is indicated. N = 4,315 cells.

B Progression of arrest states over time following etoposide treatment. Etoposide-treated cells (upper panel: colored by condition, lower panel: colored by DNA content) populating 2C, 4C, 8C and senescent arrest states at each day of treatment (1–4 days).

C Distribution of senescence-associated β-galactosidase (SA-βgal) activity in individual cells following etoposide treatment (1 μM, 0–4 days).

D–K (D) GSK3β, (E) phospho(Thr157)-p27, (F) p27, (G) CDK4, (H) cyclin D1, (I) cyclin E, (J) cell area and (K) DNA:cytoplasm ratio of etoposide-treated cells are plotted on the map. Median nuclear values are shown in D-I. Area indicated with a dotted line represents the senescent region of the map.

L–O (L) Cyclin A, (M) cyclin B1, (N) CDH1 and (O) SKP2 of etoposide-treated cells are plotted on the map. Median nuclear values are shown. Area indicated with a dotted line shows the trajectory of mitotic skipping and transition into senescence.

©2022 The Authors

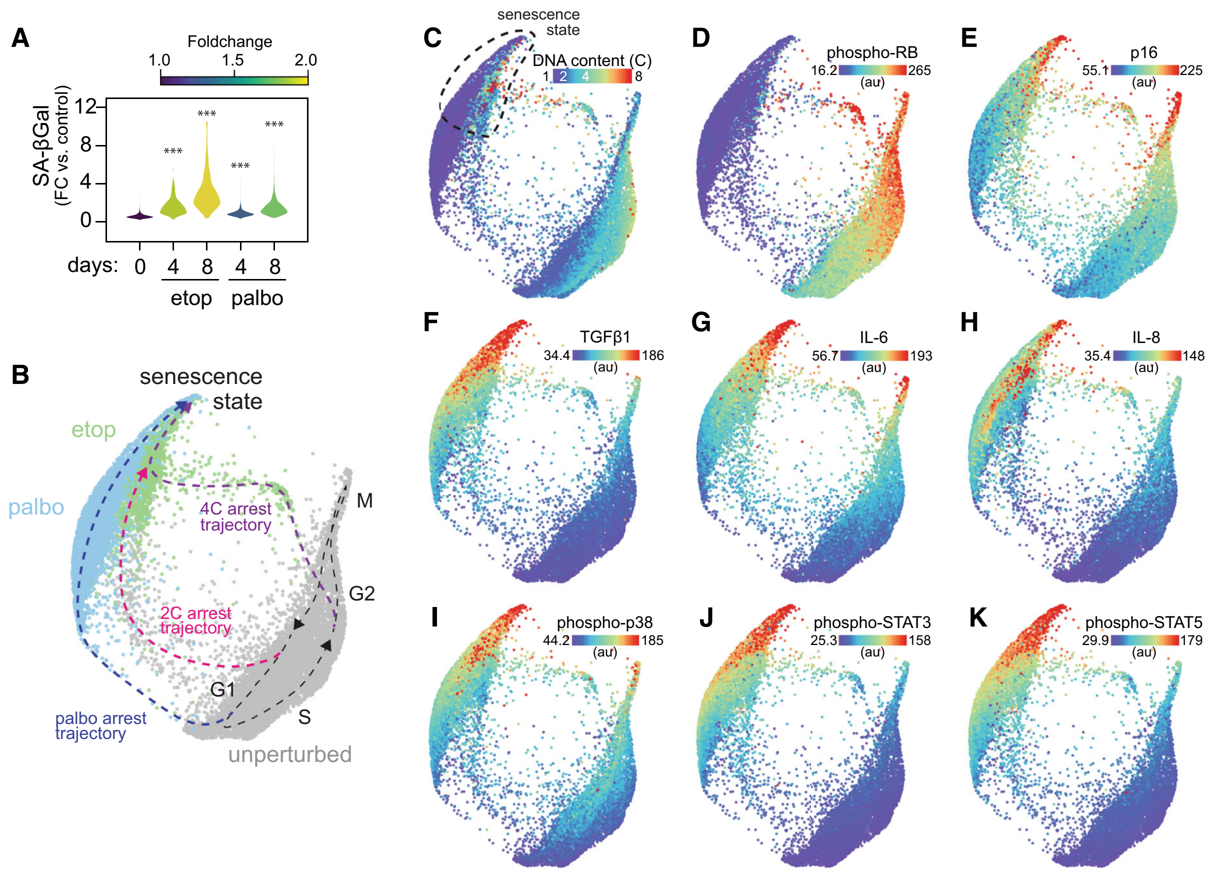

Figure 5. Palbociclib- and etoposide-induced arrest trajectories converge on a single state of cellular senescence.

A Distribution of senescence-associated β-galactosidase (SA-βgal) activity in individual cells following etoposide or palbociclib treatment (1 μM, 4/8 days). Control: N = 6,050 cells, etop 4 days: N = 409 cells, etop 8 days: N = 732 cells, palbo 4 days: N = 1837 cells, palbo 8 days: N = 1,677 cells. Statistical significance was determined using a one-way analysis of variance (ANOVA) with Sidak's *post hoc* test (***P < 0.0001).

B Unified cell cycle map of the unperturbed (gray), palbociclib- (palbo, 1 μM, 4/8 days, blue) and etoposide- (etop,1 μM, 4/8 days, green) treated cells. Control: N = 10,499 cells, etop: N = 2,692 cells, palbo: N = 4,931 cells.

C–K (C) DNA content, (D) phospho/total RB, (E) p16, (F) TGFβ1, (G) IL-6, (H) IL-8, (I) phospho-p38, (J) phospho-STAT3 and (K) phospho-STAT5 are plotted on the map. Median nuclear values are shown.

etoposide-induced cell cycle exit from both G1 and G2, while palbociclib-induced cell cycle exit from G1 along a distinct arrest trajectory (Fig 5B–D). These arrest trajectories, however, all converge on a single terminal state possessing elevated expression of the CDK inhibitors p16 and p27, SASP factors TGFβ1, IL-6 and IL-8, as well as activation of NF-κb (phospho-p65), AKT, p38, BCL-2, STAT3, STAT5 and SMAD2 (Figs 5E–K and EV5B–F).

Our results suggest that the cell cycle status of senescent cells is a G1-like molecular state. If true, then the mechanisms that maintain this arrested state and, in turn, govern the molecular routes to possible cell cycle reentry, should be limited to G1 regulatory events. Consistent with this hypothesis, we found that the upward trajectory through the senescent region of the replication stress arrest architecture (Fig 6A) showed molecular changes consistent with cell cycle reentry in G1 and progress toward S phase including sequential increases in cyclin D1 (Fig 4H), cyclin E (Fig 4I), the DNA licensing factor Cdt1 (Fig 6B), and E2F activity (Fig 6C). As previously mentioned, we also observed a gradual accumulation of polyploid cells with 8C DNA content following etoposide treatment (Figs 4A and B, and 6A). After 4 days of treatment, we observed that 5.1% of etoposide-treated cells resided in this 8C polyploid state. We therefore hypothesized that cells might be able to reenter the cell cycle from the terminal state following mitotic skipping and undergo a second round of DNA replication, or "endoreduplication" (Fox & Duronio, 2013). To validate these observations, we performed time-lapse imaging of RPE cells expressing p21-YPet as well as cell cycle (PCNA-mTq2) and CDK2 activity (DHB-mCherry) sensors for 4 days following etoposide treatment. We indeed observed cells exiting the cell cycle from G2, remaining arrested for an extended period of time (15–30 h), then reentering the cell cycle (as indicated by an increase in CDK2 activity) and transitioning into a second S phase (as indicated by an increase in PCNA foci) without first undergoing mitosis (Fig 6D). We observed this endoreduplication in five of 117 cells over 4 days of imaging, roughly the same proportion of cells that we observed in our fixed cell 4i experiment. We similarly observed the appearance of

 ©2022 The Authors

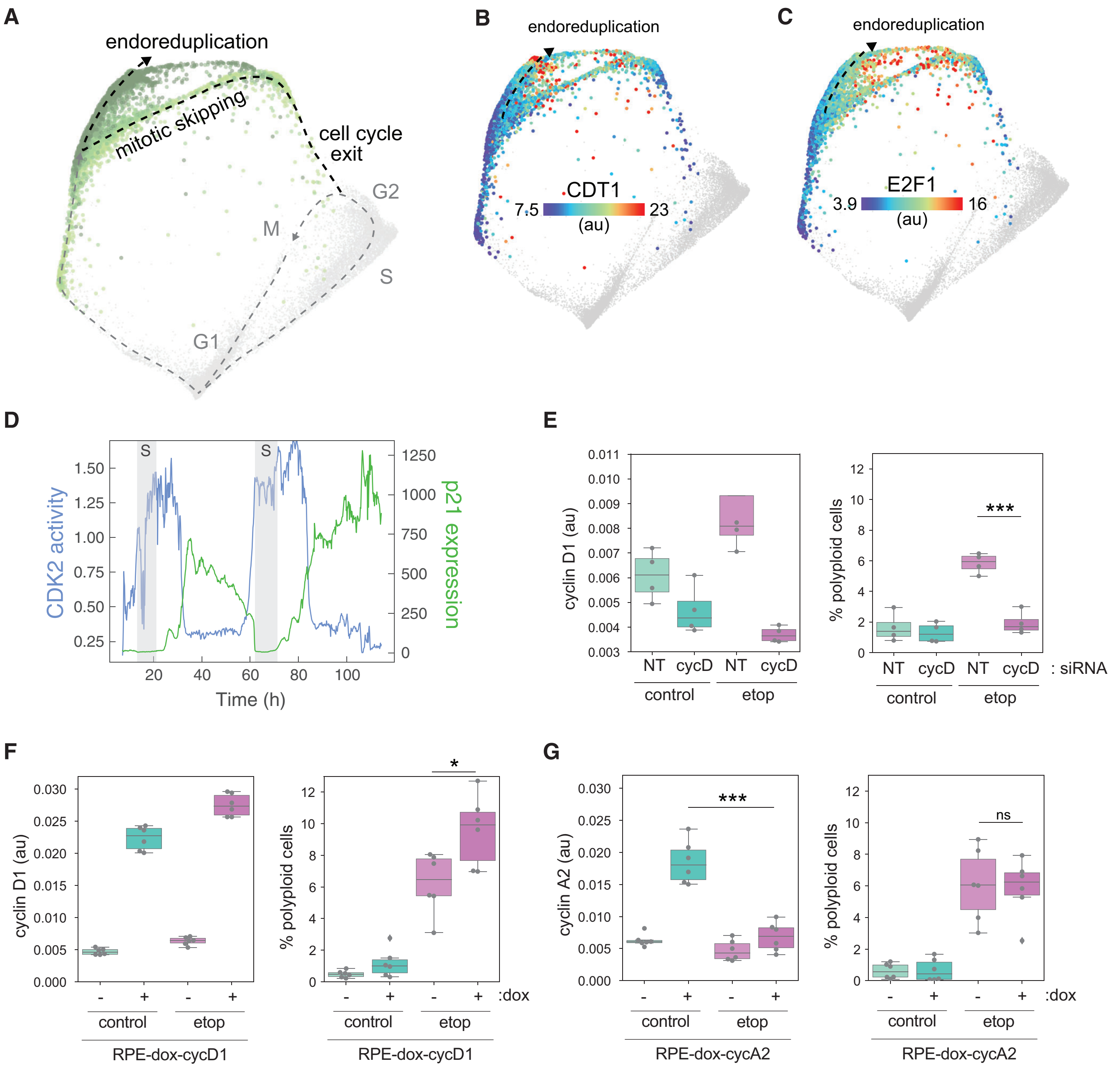

Figure 6. Sustained replication stress can induce polyploidy through mitotic skipping and endoreduplication.

A Arrest architecture of replicative stress. The trajectory of endoreduplication following cell cycle arrest and mitotic skipping indicated.

B, C (B) CDT1 and (C) E2F1 are plotted on the map. Dotted line indicates the trajectory to endoreduplication. Median nuclear values are shown.

D Representative single-cell trace demonstrating mitotic skipping and endoreduplication following etoposide treatment (1 μM) by time-lapse imaging. CDK2 activity (DHB-mCherry, blue), cell cycle phase (PCNA-mTq2, S phase shown as gray shaded area) and p21-YPet intensity (green) are plotted versus time of etoposide treatment.

E Cyclin D1 abundance (left) and the proportion of polyploid cells (right), as measured by immunofluorescence and Hoechst staining, respectively, following siRNA-mediated knockdown of cyclin D in control and etoposide-treated cells. Boxplots show data from four independent replicates (gray circles).

F Cyclin D1 abundance (left) and the proportion of polyploid cells (right), as measured by immunofluorescence and Hoechst staining, respectively, following doxycycline (dox)-induced upregulation of cyclin D1 in control and etoposide-treated cells. Boxplots show data from six independent replicates (gray circles).

G Cyclin A abundance (left) and the proportion of polyploid cells (right), as measured by immunofluorescence and Hoechst staining, respectively, following doxycycline (dox)-induced upregulation of cyclin A2 in control and etoposide-treated cells. Boxplots show data from six independent replicates (gray circles).

Data information: Statistical significance in right panels of E-G was determined using a two-way analysis of variance (ANOVA) with Sidak's *post hoc* test (****P* < 0.001, **P* = 0.02). For panels (E–H), boxes show the interquartile range, the whiskers indicate the full distribution of points and the central band represents the population.

©2022 The Authors

polyploid MCF10A cells in response to sustained replication stress (Fig EV4M).

To test whether cyclin D can drive cell cycle reentry and endoreduplication after a G2 exit (as suggested by the structure), we treated cells with siRNA against all three cyclin D isoforms following etoposide treatment. Knockdown of cyclin D completely abolished the appearance of polyploid cells after 4 days of etoposide treatment (Fig 6E). Conversely, doxycycline (dox)-induced overexpression of cyclin D1 significantly increased the number of polyploid cells (Fig 6F), confirming its role in the generation of polyploidy. These findings indicate that increased expression of G1 cyclins in senescent cells can provide a route for cells to escape from this “irreversibly” arrested state. Dox-induced expression of the S/G2 cyclin A2, on the other hand, could not increase the proportion of polyploid cells (Fig 6G, right panel). In fact, following etoposide treatment, the dox-induced expression of cyclin A2 was significantly less than control cells following dox induction (Fig 6G, left panel), consistent with the reactivation of APC/C-induced degradation of cyclin A that typically occurs during G1. Thus, regardless of the phase of cell cycle exit (and DNA content), the senescent state resembles a G1 molecular state, which may narrow the mechanisms that stabilize this cell cycle arrest—as well as those that could reverse it—to G1 regulatory events.

A map of cell cycle arrest

By projecting all three stresses onto the same structure (Fig 7A and B) and overlaying our inferred trajectories (Fig 7C), a comprehensive architecture of cell cycle arrest emerged (Fig 7A). The arrest architectures of replication and oxidative stress exhibited a high degree of similarity, notably featuring two paths of cell cycle exit from either G1 or G2. Hypomitogenic arrest, on the other hand, induced a distinct arrest architecture with cells diverging during G2 and undergoing mitosis directly into a different state of arrest with 2C DNA content. The majority of spontaneously arrested cells (unperturbed cells with low RB phosphorylation) were observed along a trajectory toward the 2C arrest state driven by an increase in p21 (Fig 1I).

Discussion

Here, we combined hyperplexed, single-cell imaging with manifold learning to visualize the molecular architecture of cell cycle arrest and obtain a better understanding of the diversity of molecular mechanisms that govern it. In response to hypomitogenic stress, cells diverged from the proliferative trajectory in G2 accompanied by a decrease in cyclin D1 abundance (Fig 2D and G). Due to this deficiency in cyclin D1, cells cannot sustain RB phosphorylation through mitosis after the G2/M cyclins are degraded (Gookin *et al*, 2017; Yang *et al*, 2017; Moser *et al*, 2018; Stallaert *et al*, 2022). Mitogenic signaling regulates global protein translation rates throughout the cell cycle to control cyclin D abundance in mother cell G2 and influence the proliferation/arrest decision of daughter cells (Min *et al*, 2020). We found that decreased mitogenic signaling through serum starvation induced a reduction in nearly all proteins measured (including cyclin D1) as cells exited the cell cycle (Fig 2H), consistent with a inhibition of global protein synthesis due to a decrease in ribosomal RNA and protein synthesis (Donati *et al*, 2011) and cap-dependent translation (Liu & Qian, 2014). The abundance of p27, on the other hand, not only resisted this downregulation but was actually increased following serum starvation, likely due to the presence of an internal ribosome entry site (IRES) in the 5′-UTR of its mRNA, permitting cap-independent translation (Millard *et al*, 1997; Miskimins *et al*, 2001; Jiang *et al*, 2007), which often allows selective translation of specific proteins in conditions of cellular stress (Sonenberg & Hinnebusch, 2009). This steady increase in p27 as cells progressed further along the hypomitogenic arrest trajectory is also consistent with a molecular state that could

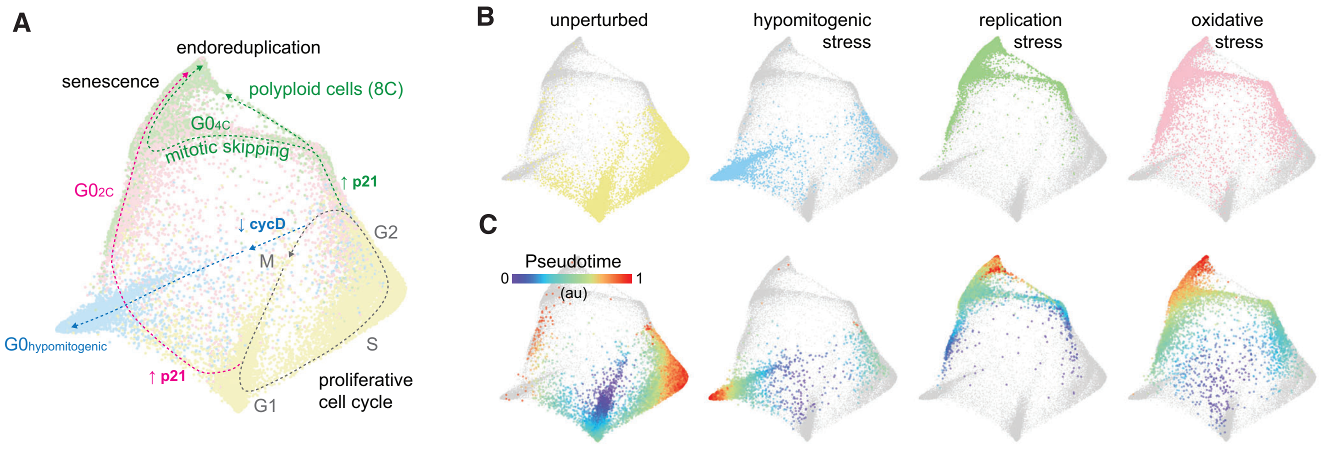

Figure 7. The overall architecture of cell cycle arrest.

A, B Summary map of cell cycle arrest. Unperturbed (yellow), serum-starved (blue), etoposide- (green) and H_2O_2-treated (pink) cells are plotted on a (A) single map. (B) Each condition is shown individually on the map (other conditions are shown in lighter gray). Inferred trajectories of the proliferative cell cycle and all arrest trajectories are shown.

C Diffusion pseudotime is plotted onto the map of each condition.

 ©2022 The Authors

increase the depth of cell cycle arrest (Binné *et al*, 2007; Fujimaki *et al*, 2019).

Following replication and oxidative stress, cells diverged from the proliferative trajectory at two distinct points: either (i) immediately following cell division or (ii) after DNA replication (Fig 3). In both scenarios, cell cycle exit is driven by a DNA damage response and p21 induction (Fig 3F). The cell fate decision between these two trajectories in response to replication stress was predicted by the timing of the p21 induction in individual cells (Fig 3F), as previously shown (Barr *et al*, 2017). We found that cells that exited from G2 (along the 4C trajectory) transitioned from a G2-to-G1-like molecular state due to mitotic skipping, in which the APC/C ubiquitin ligase is activated in the absence of cell division (Fig 4L–O). It was previously reported these mitotic skipping mechanisms can precede senescence following a G2 cell cycle exit (Wiebusch & Hagemeier, 2010; Suzuki *et al*, 2012; Johmura *et al*, 2014; Krenning *et al*, 2014; Müllers *et al*, 2014). We directly resolved this molecular trajectory on the cell cycle map and found that it converged with the arrest trajectory of cells that exited the cell cycle after cell division. Both trajectories tended toward a single senescent state with a G1-like molecular signature (Fig 4A).

We present several lines of evidence that, under these experimental conditions, senescence is an obligate G1-like molecular state. First, we found that regardless of the phase of cell cycle exit following etoposide, cells accumulate over time in a single senescent state characterized by high expression of G1 effectors and low expression of G2 effectors (Fig 4). In addition, we observed convergence on a single senescent state following treatment with palbociclib and etoposide, which induce cell cycle exit primarily from G1 and G2, respectively (Fig 5). Although this state of arrest was stable under treatment, it was not completely irreversible (Fig 6D), and cell cycle reentry could be induced by increased expression of the G1 effector cyclin D1 (Fig 6E and F). By contrast, overexpression of the S/G2 effector cyclin A2 had no effect on cell cycle reentry (Fig 6G). This G1-like molecular signature with high G1 cyclins, low G2/M cyclins, and four copies of DNA was also detected in senescent osteosarcoma (U-2 OS) cells (Fig EV4).

Our results call into question the "irreversibility" of cell cycle arrest in senescence. We found that cell cycle reentry can occur in cells that otherwise possess established hallmarks of senescence (e.g., SA-β-gal activity (Hjelmeland *et al*, 1999) and low DNA:cytoplasm ratio (Neurohr *et al*, 2019)). These reentry events are relatively rare (~ 5% of cells over 4 days of sustained replication stress) but can be induced by increased expression of G1 effectors such as cyclin D1. These results are consistent with senescent cell cycle arrest being a strong attractor state (Choi *et al*, 2012; Chong *et al*, 2018), but not irreversible. Under normal physiological conditions, this senescence attractor may be sufficiently robust to maintain cell cycle arrest during normal biochemical variability. However, the supraphysiological concentrations of mitogens in which laboratory tissue cultures are grown, which stimulate the expression of cyclin D and other proliferative factors (Lukas *et al*, 1996), may allow cells to access additional states that can escape this attractor. It is possible that oncogenic transformation, which often involves the amplification of mitogenic signaling, might also increase access to these biochemical states and allow cell cycle reentry from senescence. In addition, many cancer therapies are designed to induce irreversible cell cycle arrest through the generation of DNA damage, including radiotherapy and DNA-damaging agents such as etoposide. However, even a low rate of cell cycle reentry from this arrest state could lead to tumor recurrence. Given the role that cyclin D:CDK4/6 plays in cell cycle reentry from senescence (Fig 6E and F), it is conceivable that sequential treatment with a DNA-damaging agent followed by an FDA-approved CDK4/6 inhibitor such as palbociclib might help reduce tumor recurrence. Furthermore, whole genome duplication can facilitate tumorigenesis (Fujiwara *et al*, 2005) and is becoming an area of interest for cancer therapy (Quinton *et al*, 2021). We show that sustained replication stress, which is a hallmark of many cancers, can induce genome duplication through mitotic skipping and endoreduplication. Future studies exploring the role of this arrest trajectory in tumor initiation and progression may reveal new therapeutic strategies for the treatment of certain cancers.

Finally, we assert that senescence is an obligate G1-like state as defined by the molecular signature of cell cycle proteins, which consequently governs the mechanisms that maintain arrest (or allow escape from it). While we also observed that many signaling and SASP markers are also similar between palbociclib- and etoposide-induced senescence (Fig 5F–K), it is possible that these senescent states are distinct from one another in other features not measured here, which may generate differences in other functional aspects of the senescent state (e.g., metabolism and secretory phenotype). It is unclear whether these other functional properties of senescent cells are also reversed when they reenter the cell cycle.

Materials and Methods

Cell lines and culture

Retinal pigment epithelial cells (hTERT RPE-1, ATCC, CRL-4000) and MCF10A (ATCC, CRL-10317) were used for fixed cell experiments. The RPE-PCNA-mTq2/p21-YPet/DHB-mCherry (Stallaert *et al*, 2022) and RPE-p21-mTq2/cycD1-mVenus/DHB-mCherry/H2B-mIFP (Zerjatke *et al*, 2017) cell lines used for time-lapse imaging were previously described. RPE cells were grown at 37°C and 5% CO_2 in DMEM (Gibco, 11995-065) with 10% fetal bovine serum (FBS; Sigma, TMS-013-B), 2 mM L-glutamine (ThermoFisher Scientific, 25030081), and penicillin/streptomycin (P/S; ThermoFisher Scientific, 15140148). FluoroBrite™ DMEM (Gibco, A18967-01) supplemented with 10% FBS and 2 mM L-glutamine was for time-lapse imaging. MCF10A cells were grown at 37°C and 5% CO2 in MEGM media (Lonza, CC-3151) supplemented with MEGM Supplements (Lonza, CC-4136), 100 ng/ml cholera toxin (Millipore Sigma C8052), 5% fetal bovine serum (FBS; Sigma, TMS-013-B), and penicillin/streptomycin (P/S; ThermoFisher Scientific, 15140148). All cell lines were authenticated by STR profiling (ATCC) and confirmed to be mycoplasma free. Where indicated, cells were treated with etoposide (MedChemExpress, HY-13629), H_2O_2 (Millipore Sigma, H1009) or palbociclib (Selleckchem, S1116).

Antibodies

All antibodies used in this study were chosen using BenchSci (http://app.benchsci.com) to identify high quality, previously published/validated primary antibodies and are listed in Table EV1.

©2022 The Authors

Time-lapse imaging

Time-lapse imaging was performed as previously described (Stallaert *et al*, 2022) using a Nikon Ti Eclipse inverted microscope equipped with a Nikon Plan Apochromat Lambda 40x objective (NA = 0.95) and an Andor Zyla 4.2 sCMOS detector, using Nikon Perfect Focus System (PFS). A climate-controlled enclosure (Okolabs) was used to maintain constant temperature (37°C) and atmosphere (5% CO_2). Combinations of the following filter sets (Chroma) were used as required (excitation; beam splitter; emission filter): CFP (425–445/455/465–495 nm), YFP (490–510/515/520-550 nm), mCherry(540–580/585/593–668) and Cy5(590–650/660/663-738 nm).

RPE-PCNA-mTq2/p21-YPet/DHB-mCherry and RPE-p21-mTq2/cycD1-mVenus/DHB-mCherry/H2B-mIFP cells were imaged every 10 min and RPE-mVenus-p27K- cells were imaged every 15 min. Stitched 4 × 4 images were acquired and field illumination correction was performed before stitching. Image analysis and post-processing were performed using NIS-Elements AR software with General Analysis 3. CDK2 activity was calculated as the ratio of background corrected cytoplasmic to nuclear intensity of DHB-mCherry. The cytoplasm signal was quantified in a 15-pixel ring outside the segmented nucleus, with a 2-pixel gap between the nucleus and the ring. p21-YPet or cyclinD1-mVenus intensity was calculated as the background corrected median nuclear intensity. The appearance/disappearance of nuclear PCNA foci was used to manually annotate cell cycle phase transitions.

Image analysis was performed using Python (3.7.10) with Cellpose (v.0.6.5) (Stringer *et al*, 2021) segmentation algorithm, Scikit-image image processing library (v.0.18.2) (van der Walt *et al*, 2014) and BayesianTracker linking algorithm (v.0.4.1) (Ulicna *et al*, 2021). Errors in segmentation and tracking were corrected manually using the napari graphical interface (v.0.4.10) (Sofroniew *et al*, 2022).

Iterative indirect immunofluorescence imaging (4i)

Sample preparation was performed as previously described (Stallaert *et al*, 2022). Stitched 8 × 8 images were acquired for each condition using the Nikon Ti Eclipse microscope described above with the following filter cubes (Chroma): DAPI(383–408/425/435-485 nm), GFP(450–490/495/500-550 nm), Cy3(530–560/570/573-648 nm) and Cy5(590–650/660/663-738 nm). Images from successive rounds were aligned in Python (v3.7.1) using the StackReg library (Thévenaz *et al*, 1998) and corrected using manually selected fiduciary points if necessary. Segmentation and feature extraction were performed in CellProfiler (v3.1.8) (Stirling *et al*, 2021) using standard modules. Cell cycle phase annotations were inferred using a four-component gaussian mixture model (scikit-learn v1.1.1) trained on the median nuclear intensities of PCNA, CDH1, SKP2, cyclin A, E2F1, cyclin B1 and phospho-p27, as well as nuclear area and DNA content.

Data integration

The following normalization was used to integrate the independent datasets containing 4i measurements of (i) unperturbed (control 1), serum-starved, etoposide- and H_2O_2-treated cells and (ii) unperturbed (control 2) and palbociclib-treated cells. For each of the datasets, first the control data were z-normalized. Each treatment condition was subsequently normalized using the mean and standard deviation of its matched control, to preserve relative fold-changes induced by treatment. Bimodal features used for subsequent embedding were peak normalized (2C and 4C peaks in DNA content normalized to 2 and 4, phospho-RB and phospho/total RB hypo- and hyperphosphorylation peaks normalized to 0 and 1).

Feature selection and manifold learning

We previously trained random forest models on ground truth annotations of cell cycle phase and age to identify an optimized feature set that resolves the cell cycle manifold in RPE cells (Stallaert *et al*, 2022). This validated feature set was used to inform feature selection in the current study (Table EV2). Manifold learning was performed using Potential of Heat-diffusion for Affinity-based Transition Embedding (PHATE) (Moon *et al*, 2019) using the feature set described above as input variables. PHATE coordinates are projections of the data that locate each cell in relation to other cells based on their molecular signatures as measured by 4i. Unlike other dimensionality reduction approaches such as principle components analysis (PCA), movement along PHATE axes does not correspond to fixed, consistent changes in feature values. Instead, PHATE preserves the log-transformed probability distances between cells based on feature set. Cells that differ greatly in their features will thus have a greater potential distance and be placed further apart in the PHATE projection. PHATE was run on z-normalized features with the following parameter sets for cell cycle maps: (Fig 1B: k-nearest neighbor (knn) = 150, t = 20, gamma = 1; Fig 2A: knn = 15, t = 26, gamma = 1, Fig 3A: knn = 75, t = 19, gamma = 1, Fig 5A: knn = 50, t = 19, gamma = 1, Fig 7A: knn = 150, t = 10, gamma = 0.25, Fig EV5A: knn = 150, t = 10, gamma = 0.25, Fig EV3A: knn = 100, t = 30, gamma = 1). The phases of the proliferative cell cycle (G1/S/G2/M) in unperturbed cells were annotated manually using characteristic changes in DNA content and cyclin abundance.

Data visualization

Python (v3.7.1) and Jupyter Notebooks (v6.1.4) were used for data visualization using matplotlib (v3.3.2), seaborn (v0.11.0), and scanpy (v1.6; Wolf *et al*, 2018) libraries, as well as GraphPad Prism (v8).

siRNA

RPE cells were treated with DMSO or etoposide (1 μM) for 24 h then transfected with nontargeting (Dharmacon, D-001810-10-0) or cyclin D1/D2/D3 (SMARTPools L-003210-00-0005/L-003211-00-0005/L-003212-00-0005) siRNA pools using the DharmaFECT 1 transfection reagent (T-2001-01) as per the manufacturer's protocol and incubated for 3 days prior to fixation. Immunofluorescence was performed as per the 4i protocol described above.

Data availability

Single-cell datasets are available at Zenodo: doi:10.5281/zenodo.6394367.

©2022 The Authors

Expanded View for this article is available online.

Acknowledgments

We would like to thank Dr. Sam Wolff (UNC Chapel Hill) for support with imaging and Jamshaid Shahir for providing an intuitive explanation of PHATE coordinates. The RPE-p21-mTq2/cycD1-mVenus/DHB-mCherry/H2B-mIFP was a generous gift from Dr. Jorg Mansfield (Institute for Cancer Research, London, England) and Dr. Sabrina Spencer (University of Colorado Boulder). The pMXs-IP-mVenus-p27K− plasmid used to make the RPE-mVenus-p27K- cell line was a gift from T. Kitamura (University of Tokyo, Minato-ku, Tokyo). This work was supported by NSF CAREER Award 1845796 (JEP); NIH grants R01-GM138834 (JEP), R01-GM083024 (JGC), R01-GM102413 (JGC), and R35-GM141833 (JGC); the Chan Zuckerberg Initiative DAF (an advised fund of Silicon Valley Community Foundation 2020-225716) (KMK); JCL was supported by an HHMI Gilliam Fellowship for Advanced Study (GT10886); and NIH/NIGMS awards to UNC: R25-GM055336 and T32-GM007040.

Author contributions

Wayne Stallaert: Conceptualization; data curation; formal analysis; investigation; visualization; methodology; writing – original draft; project administration; writing – review and editing. **Sovanny R Taylor:** Investigation. **Katarzyna M Kedziora:** Data curation; software; formal analysis; methodology; writing – review and editing. **Colin D Taylor:** Software; formal analysis. **Holly K Sobon:** Investigation. **Catherine L Young:** Investigation. **Juanita C Limas:** Resources. **Jonah Varblow Holloway:** Data curation. **Martha S Johnson:** Resources; investigation. **Jeanette Gowen Cook:** Supervision; funding acquisition; writing – review and editing. **Jeremy E Purvis:** Conceptualization; resources; supervision; funding acquisition; writing – review and editing.

In addition to the CRediT author contributions listed above, the contributions in detail are:

WS and JEP conceived of the project. WS, KMK, JGC, and JEP designed the experiments. WS, HKS, SRT, and CLY performed the 4i experiments. WS, MSJ, and SRT performed the time-lapse imaging. WS, KMK, JH, MSJ, and CDT performed image analysis. MSJ and JCL created cell lines. WS wrote the manuscript with the help of all authors.

Disclosure and competing interests statement

The authors declare that they have no conflicts of interest.

References

Arora M, Moser J, Phadke H, Basha AA, Spencer SL (2017) Endogenous replication stress in mother cells leads to quiescence of daughter cells. *Cell Rep* 19: 1351–1364

Barr AR, Cooper S, Heldt FS, Butera F, Stoy H, Mansfeld J, Novak B, Bakal C (2017) DNA damage during S-phase mediates the proliferation-quiescence decision in the subsequent G1 via p21 expression. *Nat Commun* 8: 14728

Binné UK, Classon MK, Dick FA, Wei W, Rape M, Kaelin WG Jr, Näär AM, Dyson NJ (2007) Retinoblastoma protein and anaphase-promoting complex physically interact and functionally cooperate during cell-cycle exit. *Nat Cell Biol* 9: 225–232

Cameron IL, Greulich RC (1963) Evidence for an essentially constant duration of DNA synthesis in renewing epithelia of the adult mouse. *J Cell Biol* 18: 31–40

Choi M, Shi J, Jung SH, Chen X, Cho K-H (2012) Attractor landscape analysis reveals feedback loops in the p53 network that control the cellular response to DNA damage. *Sci Signal* 5: ra83

Chong KH, Zhang X, Zheng J (2018) Dynamical analysis of cellular ageing by modeling of gene regulatory network based attractor landscape. *PLoS One* 13: e0197838

Coats S, Flanagan WM, Nourse J, Roberts JM (1996) Requirement of p27Kip1 for restriction point control of the fibroblast cell cycle. *Science* 272: 877–880

Coller HA, Sang L, Roberts JM (2006) A new description of cellular quiescence. *PLoS Biol* 4: e83

Demple B, Halbrook J (1983) Inducible repair of oxidative DNA damage in *Escherichia coli*. *Nature* 304: 466–468

Donati G, Bertoni S, Brighenti E, Vici M, Treré D, Volarevic S, Montanaro L, Derenzini M (2011) The balance between rRNA and ribosomal protein synthesis up- and downregulates the tumour suppressor p53 in mammalian cells. *Oncogene* 30: 3274–3288

Fox DT, Duronio RJ (2013) Endoreplication and polyploidy: insights into development and disease. *Development* 140: 3–12

Fujimaki K, Yao G (2020) Cell dormancy plasticity: quiescence deepens into senescence through a dimmer switch. *Physiol Genomics* 52: 558–562

Fujimaki K, Li R, Chen H, Della Croce K, Zhang HH, Xing J, Bai F, Yao G (2019) Graded regulation of cellular quiescence depth between proliferation and senescence by a lysosomal dimmer switch. *Proc Natl Acad Sci U S A* 116: 22624–22634

Fujiwara T, Bandi M, Nitta M, Ivanova EV, Bronson RT, Pellman D (2005) Cytokinesis failure generating tetraploids promotes tumorigenesis in p53-null cells. *Nature* 437: 1043–1047

Glotzer M, Murray AW, Kirschner MW (1991) Cyclin is degraded by the ubiquitin pathway. *Nature* 349: 132–138

Gookin S, Min M, Phadke H, Chung M, Moser J, Miller I, Carter D, Spencer SL (2017) A map of protein dynamics during cell-cycle progression and cell-cycle exit. *PLoS Biol* 15: e2003268

Guo Y, Harwalkar J, Stacey DW, Hitomi M (2005) Destabilization of cyclin D1 message plays a critical role in cell cycle exit upon mitogen withdrawal. *Oncogene* 24: 1032–1042

Gut G, Herrmann MD, Pelkmans L (2018) Multiplexed protein maps link subcellular organization to cellular states. *Science* 361: eaar7042

Haghverdi L, Büttner M, Wolf FA, Buettner F, Theis FJ (2016) Diffusion gaussian robustly reconstructs lineage branching. *Nat Methods* 13: 845–848

Hjelmeland LM, Cristofolo VJ, Funk W, Rakoczy E, Katz ML (1999) Senescence of the retinal pigment epithelium. *Mol Vis* 5: 33

Howard A, Pelc SR (1951) Nuclear incorporation of P32 as demonstrated by autoradiographs. *Exp Cell Res* 2: 178–187

Jiang H, Coleman J, Miskimins R, Srinivasan R, Miskimins WK (2007) Cap-independent translation through the p27 5′-UTR. *Nucleic Acids Res* 35: 4767–4778

Johmura Y, Shimada M, Misaki T, Naiki-Ito A, Miyoshi H, Motoyama N, Ohtani N, Hara E, Nakamura M, Morita A *et al* (2014) Necessary and sufficient role for a mitosis skip in senescence induction. *Mol Cell* 55: 73–84

Klosinska MM, Crutchfield CA, Bradley PH, Rabinowitz JD, Broach JR (2011) Yeast cells can access distinct quiescent states. *Genes Dev* 25: 336–349

Krenning L, Feringa FM, Shaltiel IA, van den Berg J, Medema RH (2014) Transient activation of p53 in G2 phase is sufficient to induce senescence. *Mol Cell* 55: 59–72

©2022 The Authors

Kwon JS, Everetts NJ, Wang X, Wang W, Della Croce K, Xing J, Yao G (2017) Controlling depth of cellular quiescence by an Rb-E2F network switch. *Cell Rep* 20: 3223 – 3235

Liu B, Qian S-B (2014) Translational reprogramming in cellular stress response. *Wiley Interdiscip Rev RNA* 5: 301 – 315

Lukas J, Bartkova J, Bartek J (1996) Convergence of mitogenic signalling cascades from diverse classes of receptors at the cyclin D-cyclin-dependent kinase-pRb-controlled G1 checkpoint. *Mol Cell Biol* 16: 6917 – 6925

Marescal O, Cheeseman IM (2020) Cellular mechanisms and regulation of quiescence. *Dev Cell* 55: 259 – 271

Marthandan S, Priebe S, Hemmerich P, Klement K, Diekmann S (2014) Long-term quiescent fibroblast cells transit into senescence. *PLoS One* 9: e115597

Millard SS, Yan JS, Nguyen H, Pagano M, Kiyokawa H, Koff A (1997) Enhanced ribosomal association of p27(Kip1) mRNA is a mechanism contributing to accumulation during growth arrest. *J Biol Chem* 272: 7093 – 7098

Min M, Spencer SL (2019) Spontaneously slow-cycling subpopulations of human cells originate from activation of stress-response pathways. *PLoS Biol* 17: e3000178

Min M, Rong Y, Tian C, Spencer S (2020) Temporal integration of mitogen history in mother cells controls proliferation of daughter cells. *Science* 368: 1261 – 1265

Miskimins WK, Wang G, Hawkinson M, Miskimins R (2001) Control of cyclin-dependent kinase inhibitor p27 expression by cap-independent translation. *Mol Cell Biol* 21: 4960 – 4967

Moon KR, van Dijk D, Wang Z, Gigante S, Burkhardt DB, Chen WS, Yim K, van den Elzen A, Hirn MJ, Coifman RR *et al* (2019) Visualizing structure and transitions in high-dimensional biological data. *Nat Biotechnol* 37: 1482 – 1492

Morgan DO (2007) *The cell cycle: principles of control*. London: New Science Press

Moser J, Miller I, Carter D, Spencer SL (2018) Control of the restriction point by Rb and p21. *Proc Natl Acad Sci U S A* 115: E8219 – E8227

Müllers E, Silva Cascales H, Jaiswal H, Saurin AT, Lindqvist A (2014) Nuclear translocation of cyclin B1 marks the restriction point for terminal cell cycle exit in G2 phase. *Cell Cycle* 13: 2733 – 2743

Neurohr GE, Terry RL, Lengefeld J, Bonney M, Brittingham GP, Moretto F, Miettinen TP, Vaites LP, Soares LM, Paulo JA *et al* (2019) Excessive cell growth causes cytoplasm dilution and contributes to senescence. *Cell* 176: 1083 – 1097.e18

Overton KW, Spencer SL, Noderer WL, Meyer T, Wang CL (2014) Basal p21 controls population heterogeneity in cycling and quiescent cell cycle states. *Proc Natl Acad Sci U S A* 111: E4386 – E4393

Owen TA, Soprano DR, Soprano KJ (1989) Analysis of the growth factor requirements for stimulation of WI-38 cells after extended periods of density-dependent growth arrest. *J Cell Physiol* 139: 424 – 431

Quinton RJ, Di Domizio A, Vittoria MA, Kotýnková K, Ticas CJ, Patel S, Koga Y, Vakhshoorzadeh J, Hermance N, Kuroda TS *et al* (2021) Whole-genome doubling confers unique genetic vulnerabilities on tumour cells. *Nature* 590: 492 – 497

Sagot I, Laporte D (2019) Quiescence, an individual journey. *Curr Genet* 65: 695 – 699

Smith JA, Martin L (1973) Do cells cycle? *Proc Natl Acad Sci U S A* 70: 1263 – 1267

Sobotta MC, Barata AG, Schmidt U, Mueller S, Millonig G, Dick TP (2013) Exposing cells to H_2O_2: a quantitative comparison between continuous low-dose and one-time high-dose treatments. *Free Radic Biol Med* 60: 325 – 335

Sofroniew N, Lambert T, Nunez-Iglesias J, Evans K, Bokota G, Bussonnier M, Peña-Castellanos G, Winston P, Yamauchi K, Pop DD *et al* (2022) napari/napari: 0.4.15 (v0.4.15). *Zenodo* https://doi.org/10.5281/zenodo.6344271

Sonenberg N, Hinnebusch AG (2009) Regulation of translation initiation in eukaryotes: mechanisms and biological targets. *Cell* 136: 731 – 745

Sousa-Victor P, Gutarra S, García-Prat L, Rodriguez-Ubreva J, Ortet L, Ruiz-Bonilla V, Jardí M, Ballestar E, González S, Serrano AL *et al* (2014) Geriatric muscle stem cells switch reversible quiescence into senescence. *Nature* 506: 316 – 321

Spencer SL, Cappell SD, Tsai FC, Overton KW, Wang CL, Meyer T (2013) The proliferation-quiescence decision is controlled by a bifurcation in CDK2 activity at mitotic exit. *Cell* 155: 369 – 383

Stallaert W, Kedziora KM, Taylor CD, Zikry TM, Ranek JS, Sobon HK, Taylor SR, Young CL, Cook JG, Purvis JE (2022) The structure of the human cell cycle. *Cell Syst* 13: 230 – 240.e3

Stirling DR, Swain-Bowden MJ, Lucas AM, Carpenter AE, Cimini BA, Goodman A (2021) CellProfiler 4: improvements in speed, utility and usability. *BMC Bioinformatics* 22: 433

Stringer C, Wang T, Michaelos M, Pachitariu M (2021) Cellpose: a generalist algorithm for cellular segmentation. *Nat Methods* 18: 100 – 106

Sun D, Buttitta L (2017) States of G0 and the proliferation-quiescence decision in cells, tissues and during development. *Int J Dev Biol* 61: 357 – 366

Suzuki M, Yamauchi M, Oka Y, Suzuki K, Yamashita S (2012) Live-cell imaging visualizes frequent mitotic skipping during senescence-like growth arrest in mammary carcinoma cells exposed to ionizing radiation. *Int J Radiat Oncol Biol Phys* 83: e241 – e250

Thévenaz P, Ruttimann UE, Unser M (1998) A pyramid approach to subpixel registration based on intensity. *IEEE Trans Image Process* 7: 27 – 41

Ulicna K, Vallardi G, Charras G, Lowe AR (2021) Automated deep lineage tree analysis using a Bayesian single cell tracking approach. *Front Comp Sci* 3: 734559

van der Walt S, Schönberger JL, Nunez-Iglesias J, Boulogne F, Warner JD, Yager N, Gouillart E, Yu T, Scikit-Image Contributors (2014) Scikit-image: image processing in python. *PeerJ* 2: e453

Wang X, Fujimaki K, Mitchell GC, Kwon JS, Della Croce K, Langsdorf C, Zhang HH, Yao G (2017) Exit from quiescence displays a memory of cell growth and division. *Nat Commun* 8: 321

Wiebusch L, Hagemeier C (2010) p53- and p21-dependent premature APC/C-Cdh1 activation in G2 is part of the long-term response to genotoxic stress. *Oncogene* 29: 3477 – 3489

Wolf FA, Angerer P, Theis FJ (2018) SCANPY: large-scale single-cell gene expression data analysis. *Genome Biol* 19: 15

Yang HW, Chung M, Kudo T, Meyer T (2017) Competing memories of mitogen and p53 signalling control cell-cycle entry. *Nature* 549: 404 – 408

Zerjatke T, Gak IA, Kirova D, Fuhrmann M, Daniel K, Gonciarz M, Müller D, Glauche I, Mansfeld J (2017) Quantitative cell cycle analysis based on an endogenous all-in-one reporter for cell tracking and classification. *Cell Rep* 19: 1953 – 1966

License: This is an open access article under the terms of the Creative Commons Attribution License, which permits use, distribution and reproduction in any medium, provided the original work is properly cited.

Expanded View Figures

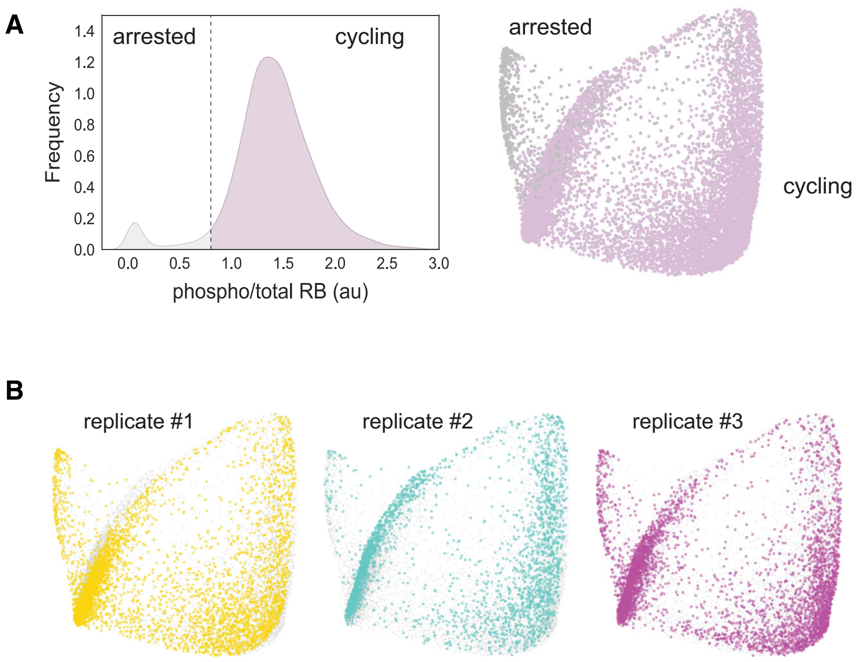

Figure EV1. The cell cycle map of unperturbed RPE cells.

A *Left*: Distribution of nuclear intensity ratios of phospho/total RB in unperturbed RPE cells. A threshold value of 0.7 was used to label cells as arrested (low phospho/total RB) or actively cycling (high phospho/total RB). *Right*: Cycling and arrested labels are overlaid on the cell cycle map.

B Cells from three technical replicates are labeled on the cell cycle map.

Figure EV2. Arrest trajectories following replication stress.

A Cell cycle map arrest of unperturbed (gray) and etoposide-treated cells (1 μM; 1 day: light green, 2 days: green, 3 days: dark green, 4 days: darker green). The unperturbed cell cycle (dotted gray line) and two arrest trajectories (into 2C and 4C, pink and purple, respectively) are indicated on the map.

B–E (B) Phospho-H2AX, (C) phospho-CHK1, (D) phospho-p65 and (E) p53 of unperturbed (left panels) or etoposide-treated cells (right panels) are plotted on the arrest architecture. Median nuclear values are shown.

F, G Heatmap of feature intensity along the (F) 2C and (G) 4C arrest trajectories. Features were ordered by hierarchical clustering according to their dynamics along each arrest trajectory. Diffusion pseudotime values were binned and pseudotime values with < 15 cells were excluded from the visualization.

H *Top*: Nuclear phospho-RB intensities of control and etoposide-treated MCF10A cells (7 days, 1 μM). Dashed-red box indicates arrested etoposide-treated cells (phospho-RB < 500 au). *Bottom*: Distribution of DNA content in arrested etoposide-treated cells. Arrested MCF10A cells were observed with both 2C and 4C DNA content.

▶

© 2022 The Authors

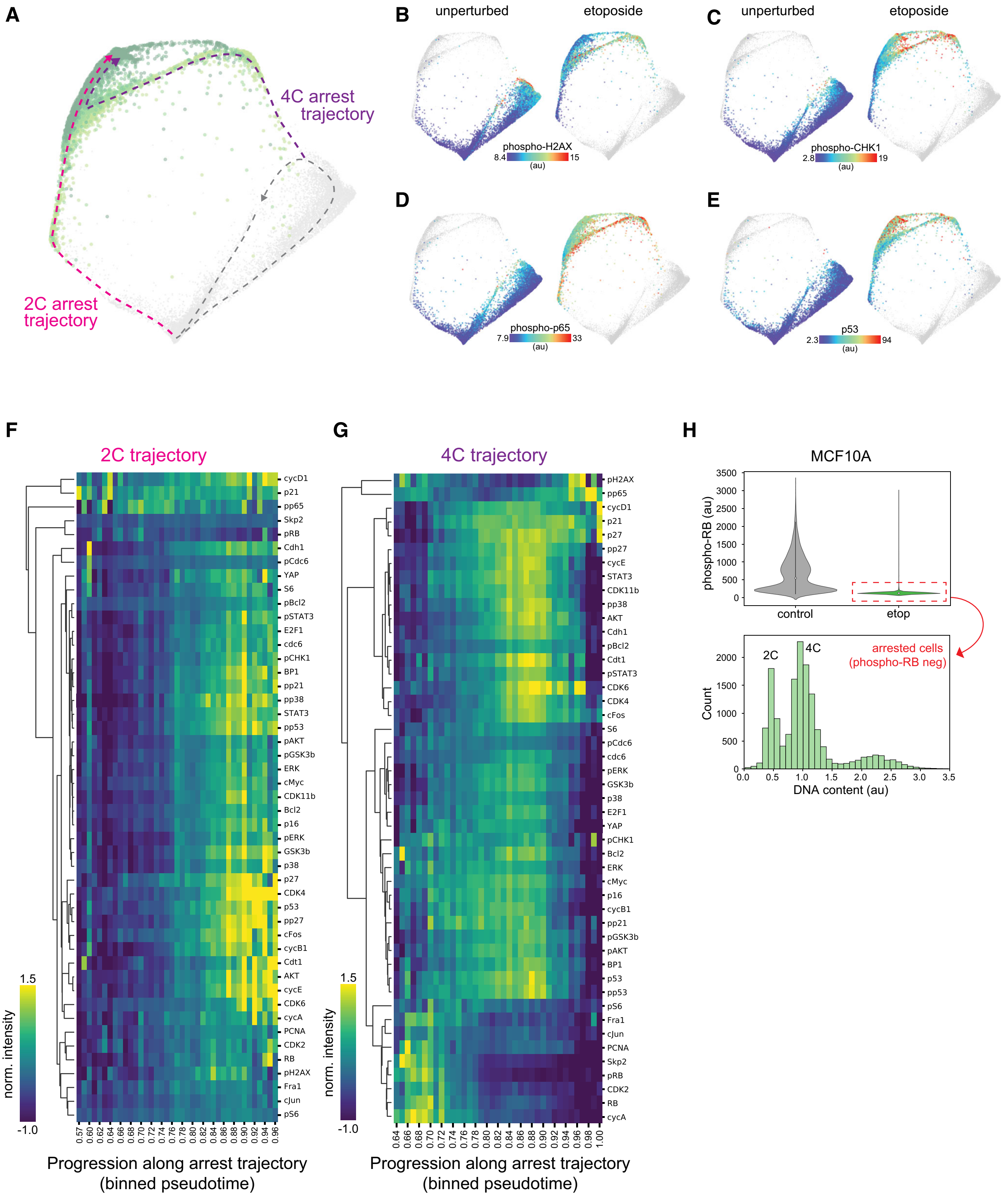

Figure EV2.

 ©2022 The Authors

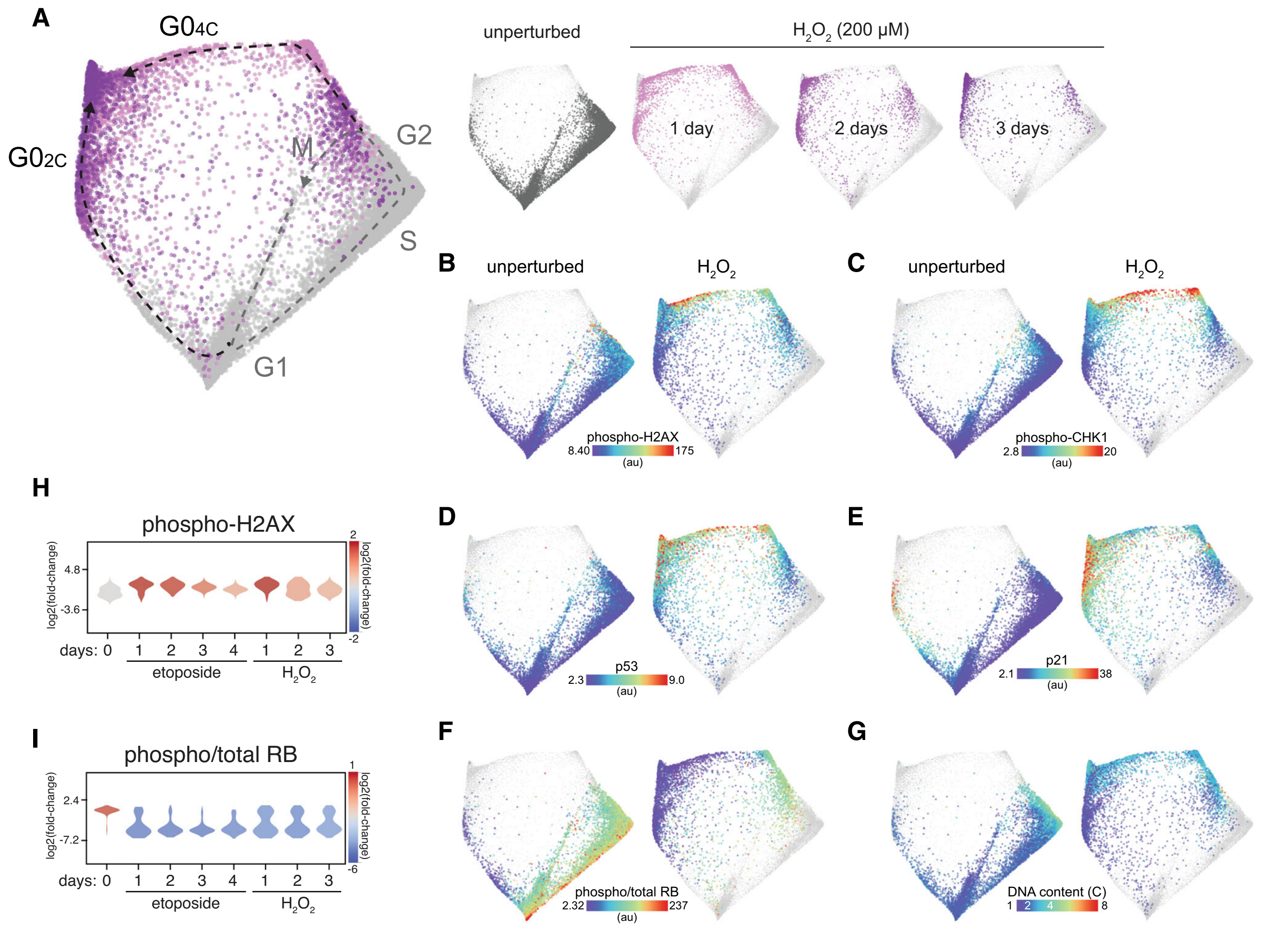

Figure EV3. The arrest architecture of oxidative stress.

A Unified cell cycle map arrest of unperturbed (gray) and H_2O_2-treated cells (200 µM; 1 day: light purple, 2 days: purple, 3 days: dark purple – see inset, *N* = 5,015 cells). The unperturbed cell cycle (dotted gray line) and two arrest trajectories (into $G0_{2C}$ and $G0_{4C}$; black dotted lines) are indicated on the map. Inset: Each condition is shown individually on the map (other conditions are shown in lighter gray).

B–G (B) Phospho-H2AX, (C) phospho-CHK1, (D) p53, (E) p21, (F) phospho/total RB and (G) DNA content of unperturbed (left panels) or H_2O_2-treated cells (right panels) are plotted on the arrest architecture. Median nuclear values are shown for B-F.

H, I Distribution of (H) phospho-H2AX and (I) phospho/total RB in individual cells following etoposide (1 µM) or H_2O_2 treatment (200 µM).

© 2022 The Authors

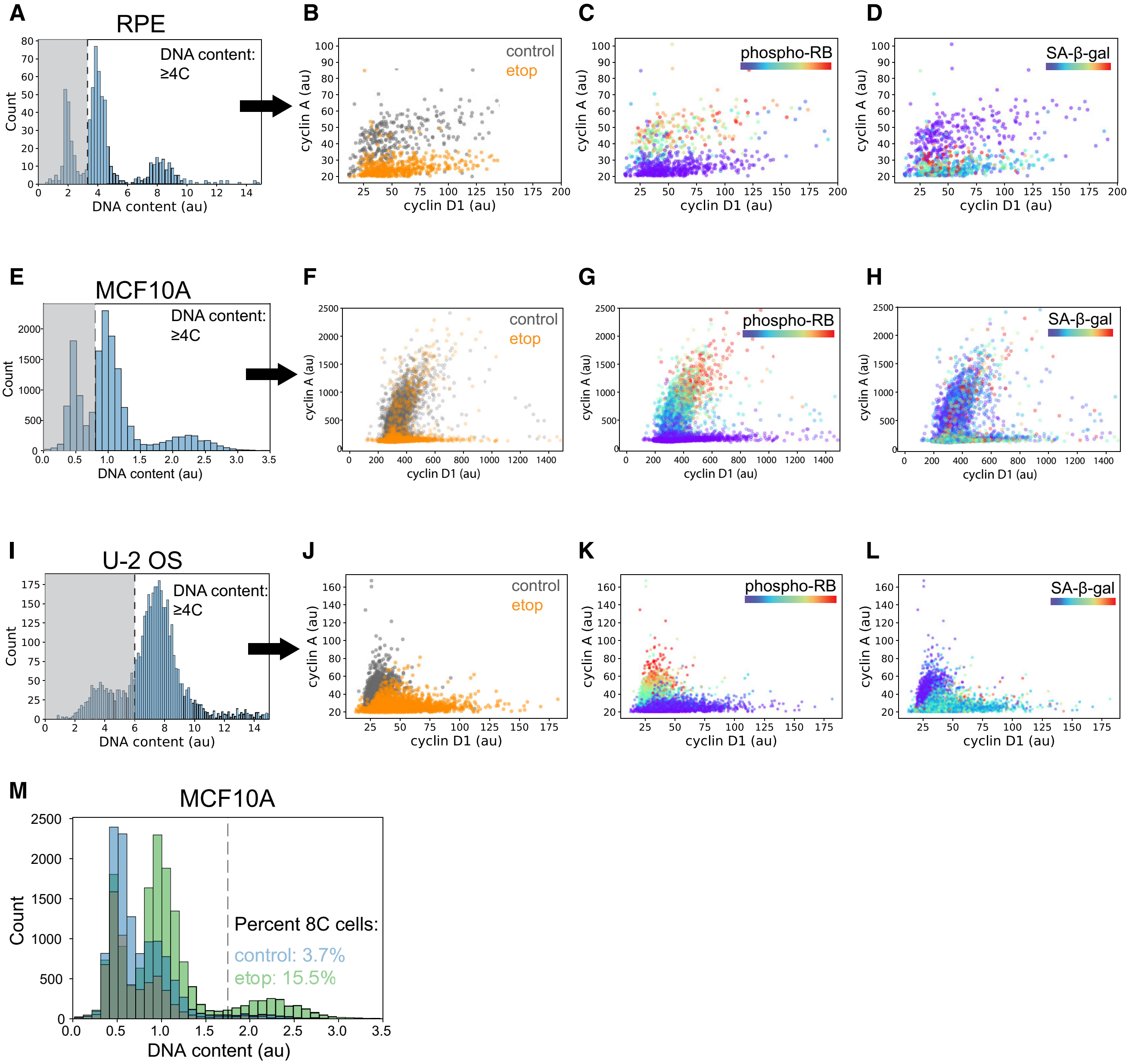

Figure EV4. Replication stress induces mitotic skipping in RPE, MCF10A and U-2 OS cells.

A–L (A) RPE cells with ≥4C DNA content were selected using the intensity of nuclear Hoechst staining. Cyclin A versus cyclin D1 intensity was plotted for each cell and overlaid with (B) condition labels (control vs. etoposide), (C) phospho-RB intensity and (D) senescence-associated β-galactosidase (SA-βgal) activity. The same analysis as above was performed on (E–H) MCF10A and (I–L) U-2 OS cells.

M Distribution of DNA content in control (blue) and etoposide-treated (green, 7 days, 1 μM) MCF10A cells. The percent of polyploid cells with DNA content = 8C is shown for both conditions.

 ©2022 The Authors

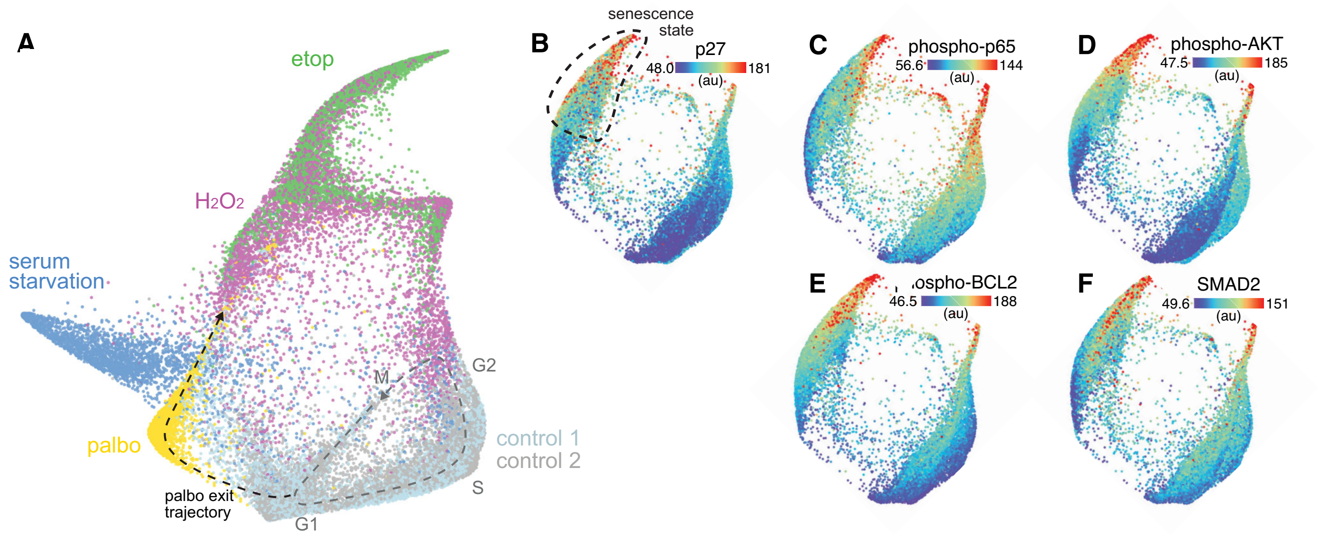

Figure EV5. The arrest architecture of palbociclib-induced arrest.

A Unified cell cycle map of unperturbed (control 2, gray) and palbociclib-treated cells (gold) from a separate experiment, plotted with unperturbed (control 1: light blue, from original experiment), serum-starved (blue) etoposide- (etop, green) and H_2O_2-treated (magenta) cells from initial experiment. Data integration is described in Materials and Methods.

B–F (B) p27, (C) phospho-p65, (D) phospho-AKT, (E) phospho-BCL2 and (F) SMAD2 are plotted on the map. Median nuclear values are shown. Dotted area indicates senescent region.

© 2022 The Authors
