# Supplementary material for: The molecular architecture of cell cycle arrest
Source: Mol Syst Biol. 2022 Sep 26;18(9):e11087. doi: 10.15252/msb.202211087 (PMC9511499; doi:10.15252/msb.202211087)
Supplement: Supplementary file 5 [file MSB-18-e11087-s002.pdf]

molecular systems biology

The molecular architecture of cell cycle arrest

Wayne Stallaert, Sovanny Taylor, Katarzyna Kedziora, Colin Taylor, Holly Sobon, Catherine Young, Juanita Limas, Jonah Varblow Holloway, Martha Johnson, Jeanette Cook, and Jeremy Purvis
DOI: 10.15252/msb.202211087

Corresponding author(s): Jeremy Purvis (jeremy_purvis@med.unc.edu)

Review Timeline:

Submission Date:	22nd Apr 22
Editorial Decision:	24th May 22
Revision Received:	27th Jul 22
Editorial Decision:	1st Sep 22
Revision Received:	7th Sep 22
Accepted:	9th Sep 22

Editor: Maria Polychronidou

Transaction Report:

(Note: With the exception of the correction of typographical or spelling errors that could be a source of ambiguity, letters and reports are not edited. Depending on transfer agreements, referee reports obtained elsewhere may or may not be included in this compilation. Referee reports are anonymous unless the Referee chooses to sign their reports.)

RE: MSB-2022-11087, The molecular architecture of cell cycle arrest

Thank you again for submitting your work to Molecular Systems Biology. We have now heard back from the three reviewers who agreed to evaluate your study. As you will see below, the reviewers think that the study presents a potentially relevant resource. However, they raise a series of concerns, which we would ask you to address in a revision.

The comments of the referees are rather clear and I think that there is no need to repeat them here. As you will see, reviewers #2 and #3 are concerned that the overall novelty of the main conclusions seems modest in view of your previous work (Stallaert et al, 2021). Related to this, reviewer #3 points out that providing evidence for the broader relevance of the conclusions by including data from an additional cell line would strengthen the study. We would also ask you to ensure that the advance/novelty of the study is clarified and presented in a balanced manner. All issues raised by the referees would need to be satisfactorily addressed. Please let me know in case you would like to discuss in further detail any of the issues raised, I would be happy to schedule a call.

On a more editorial level, we would ask you to address the following points:

Reviewer #1:

In this paper the authors characterize the molecular architecture of cell cycle arrest induced using various stressors. By utilizing iterative immunofluorescence imaging the authors measure 49 cell cycle effectors in single RPE-1 cells in different cellular compartments and combine these measurements with measurements of cell morphology to generate a list of 2952 single cell features. They utilize dimensionality reduction to project this high-dimensional data into a 2D representation of a cell cycle 'map'. Using this map the authors identify points at which cells exit the cell cycle in response to different stresses and identify molecular mechanisms governing the decision to exit the cell cycle. This nicely builds upon the author's previous work which characterized the molecular structure of the mammalian cell cycle in unperturbed cells.

Overall the work presented by the authors is of a high standard, the data are believable and the conclusions are sound, and the paper will certainly be a useful resource for researchers in the field of cell biology, specifically related to cell cycle, single cell analysis, and DNA damage. However, I wanted to raise several concerns about the text which should be addressed prior to publication.

Main Points:

1. In Figure 1C the authors show measurements of phospho/total Rb projected onto the cell cycle map. They then define the proliferative trajectory and arrest trajectory based on cells being phospho-Rb positive and negative, respectively. However, it's not clear to me how the ratio of phospho/total Rb was used to identify populations of phospho-Rb negative and positive cells. I would recommend a clearer explanation of how these two populations were defined, i.e. was a threshold used? Could this threshold be indicated on the figure to aid the reader's understanding?
Also in Figure 1C the levels of phospho/total Rb appear to be highest in G1 phase and then decline to an intermediate level as cells progress through S/G2/M phases. Given that Rb is hyperphosphorylated in G1 phase by CDK4/6 and CDK2 and that this phosphorylation is maintained through the cell cycle by these kinases, could the authors comment on why the level of Rb phosphorylation diminishes through S/G2/M phases?

2. The authors claim that: "Trajectory inference by diffusion pseudotime revealed that serum-starved cells diverge from the proliferative cell cycle during G2 (Fig 2B)." I find this statement a little confusing. Would it be possible to provide a more detailed explanation of this method and how the authors used it to arrive at this conclusion?

3. In Figure 2A-B the authors annotate the cell cycle trajectory map indicating G2 phase cells occupy the top right corner, with mitotic cells indicated to the left of these G2 cells. In subsequent panels, Figure 2D-E,I the authors depict G2 phase cells in a different location on the map, appearing in the location where mitotic cells are defined in Figure 2A-B. The Figure legend provides no description of these annotations. A clearer description of how these populations of cells are defined, and why the location of G2 cells specifically appears to change in the different figure panels would be helpful.

4. The authors state "While unperturbed cells exhibited a clear increase in cyclin D1 during G2, serum starvation significantly reduced the induction of cyclin D1 during G2, leading to less inherited cyclin D1 in daughters cells (Fig 2G) (Guo et al, 2005)." It is clear that serum starvation reduced the levels of Cyclin D in both mother and daughter cells during G2 phase compared to control cells. However, no statistical test is provided to demonstrate that this difference is indeed significant. There is no description of what the solid line and shaded region represent in this figure panel. Could a statistical test be performed to qualify the statement that the difference is indeed significant? Perhaps by calculating a fold-change in Cyclin D levels during G2 phase in control and serum starved cells?
In addition, previous work (PMID: 28869970) has identified that loss of mitogen signalling in the mother cell leads to a reduction in Cyclin D mRNA which is inherited by daughter cells, leading to lower levels of Cyclin D in daughters. Therefore, the reduced levels of Cyclin D observed in daughter cells after serum starvation could be due to a combined effect of downregulated Cyclin D protein in mother cells, which is inherited by daughter cells, as well as less Cyclin D mRNA inherited by daughter cells.

5. There are several places in the text where the authors describe a piece of data from a figure panel followed by a reference to another paper. These references seem out of context to me, since the text preceding these references only describes results from the current paper and makes no link to the work described in the cited papers. Here are a few examples:

a. "In the unperturbed cell cycle, cyclin D1 increased in late G2 and remained elevated during mitosis and after cell division (Fig 2D, left panel) (Gookin et al, 2017)."
b. "While unperturbed cells exhibited a clear increase in cyclin D1 during G2, serum starvation significantly reduced the induction of cyclin D1 during G2, leading to less inherited cyclin D1 in daughters cells (Fig 2G) (Guo et al, 2005)."
c. "...we confirmed that p27 increased steadily in individual cells following serum starvation, starting immediately after cell division (Fig 2J) (Coats et al, 1996)."

It's not clear to me what point the authors are making that are meant to be supported by these references. I assume it's because these other papers have shown similar results. If that's the case, I would recommend making that clearer by adding to the text a statement like "....as shown previously (reference)."
In general, more care could be taken throughout the manuscript to more explicitly state whether the findings in this manuscript are similar to or contradictory to previous results and to credit these previous studies appropriately.

6. In Figure 2H and I, the authors find that p27 levels are elevated in serum starved cells. The authors then state they used the mVenus-p27K- biosensor to confirm that p27 levels steadily rise after serum starvation in Figure 2J. However, the data presented in Figure 2J cannot be used to support that conclusion. The mVenus-p27K- biosensor was not designed to reflect endogenous p27 levels, but rather was originally designed as a qualitative marker of G0. Its expression is under the control of a constitutive promoter and that is the primary reason why the authors find its levels increasing linearly after the mitosis following

serum starvation. It does not reflect the transcriptional control of endogenous p27 as implied by the authors. In fact, in the Discussion section, the authors even state that p27 is primarily regulated through its 5'UTR of the mRNA, which would clearly be lacking in the mVenus-p27K- biosensor. I would recommend they remove this figure. Their data in Figure 2H and I can be used to support their statement that p27 levels were higher in serum starved cells and its levels increased with pseudotime.

7. The area of the map in Figure 4A that is labeled Terminal State appears to be labeled with Endoreduplication State in Figure 6A. These two states seem like they would be mutually exclusive, since one term implies a lack of cycling and the other term implies cells are cycling (just not dividing). A clearer reasoning behind the annotation of these two maps is needed. Its not clear why these two states clustered together when I would think they would be quite different states. The authors suggest that perhaps a small fraction of cells is escaping the Terminal State to begin endoreduplicating. However the number of cells in the endoreduplication arm appears to be much greater than the <5% of cells that were observed to escape the Terminal Phase (Fig 6D). An alternative explanation could also be that these are simply all Endoreduplicating cells. Perhaps the authors could include some single-cell traces of CDK2 activity and p21 expression for Terminally State cells after Etoposide treatment to better support these conclusions. I would expect terminal state cells to have low CDK2 activity and high p21 levels until at least 4 days after Etoposide treatment.

8. The authors state "Thus, regardless of the phase of cell cycle exit (and DNA content), the senescent state resembles a G1-like molecular state, which narrows the mechansims that stabilize this cell cycle arrest-as well as those that could reverse it-to G1 regulatory events". The authors support this statement by trying only 2 things to reverse the senescent state: over-expressing Cyclin D or Cyclin A2. This is far from an extensive test of possible reverse mechanisms. Thus, I would recommend the authors tone down this claim and amend the text such as "....which potentially narrows the mechanisms that stabilize this cell cycle arrest....", or something similar.

Minor Points:
1. In figure 2G, its not clear when the cells were serum starved relative to the data on the axis. It would be helpful to include that information in the figure legend.
2. The authors use terms such as "G0-Like state", "G2-like state" (section on Replication stress), "pseudo-G1 molecular state" (section on senescence), and "terminal state". None of these states are very well defined in the text. I would recommend more clearly defining what is meant by each term and how they are different from each other. In the case of G0-like vs Pseudo-G1, it sounds like the terms could be interchangeable. In which case, it would probably more clear for the reader to choose one term. If they are not interchangeable, then it should be stated more clearly what the difference between these two states are.

3. In the text, the authors state they stained for 49 cell cycle proteins. However, in figure 1A, the schematic says they stained 50 proteins. Furthermore, in the Extended Data Table EV1, there are only 45 proteins listed along with 2 morphological parameters. In Figure 2H and Figure EV2F,G, the heatmaps includes 47 proteins. Thus, there is an inconsistency throughout the manuscript and figures about how many proteins were actually stained for in the 4i experiments. I would recommend the authors recheck the manuscript to make it more clear how many and which proteins were stained for in each experiment.

4. The authors state: "Over several days of etoposide treatment cells proceed further along the 2C and 4C arrest trajectories, transitioning through additional molecular states (Fig 3A inset), which we will discuss in greater detail below." When looking at the inset in Fig3A it's not clear what the "additional molecular states" are that the cells transition through. Perhaps some annotation to highlight these "additional molecular states" would be useful to aid the reader's interpretation of the data.
5. There is a typo in results section when referring to loss of CDH1 and SKP2 during senescence. The figure reference is to "(Fig N-O)". Presumably this should be "(Fig 4N-O)"

Reviewer #2:

In this manuscript, Stallaert and coworkers have used hyperplexed imaging of 49 cell cycle effectors and manifold learning to obtain a map of the molecular architecture that governs cell cycle exit and progression into reversible ("quiescent") and irreversible ("senescent") arrest states. They found different paths of cell cycle arrest, specific to the types of applied stress.

This work extends recently published work by the authors (The structure of the human cell cycle - Cell Systems - 2021) by visualizing and interpreting in more detail the ways cells can go into cell cycle arrest in response to different stress signals. The manuscript is well written and interesting to read, clearly visualizing different trajectories leading to cell cycle arrest, including the molecular signatures along those trajectories.

Overall, the analyses and presented results deserve to be published somewhere. However, I remain on the fence about whether the findings fulfill the requirement of "conceptual novelty" required for publication in Molecular Systems Biology. In their recent Cell Systems paper, the authors have already presented the methodology and visualization method of the cell cycle, characterized the mechanisms driving cell cycle exit and re-entry, analyzed molecular signatures of cell cycle arrest and senescence, and even showed different cell cycle arrest trajectories (towards senescence) from both G1 and G2. Therefore, the last sentence of the abstract claiming that this work "provides a first glimpse of the overall organization of cell proliferation and

arrest" seems to be overselling the manuscript as much has already been published in their 2021 Cell Systems paper.

Apart from this main concern of conceptual novelty, here below several other questions that need to be addressed, likely possible by re-analyzing existing data, rewriting parts of the paper, and/or carrying out a few similar additional experiments.

1. Machine learning.

One common criticism of some machine-learning approaches is that they are often used in recent times to re-discover known biology. Such confirmation is in fact already interesting. Ideally though, machine-learning approaches also uncover some new biology and suggest future experiments to test these findings/suggestions. The authors already discuss both aspects, but I think it could be more clearly delineated which findings are new vs. what was known already, and how their new findings trigger new experiments/questions.

I also believe it would be good to spend some time to explain in basic terms what "manifold learning" and the PHATE visualization are. By (partially) de-mystifying such machine-learning approaches, I think readers will be able to better understand the results. Moreover, while the visualization is nice to see qualitative differences in the sense that different stresses lead to different trajectories, I wonder whether the authors could go a bit further in interpreting/explaining these denoised lower-dimensional embeddings (see next comment)?

2. PHATE visualization and interpretation.

How should we interpret the coordinates in the lower dimensional representation using PHATE? In a typical PCA, the coordinates are just a linear combination of the original coordinates, allowing to directly interpret the meaning of each principal component. Can we do something similar in PHATE? If so, could you use this to clarify your plots and learn something new (e.g. indicating what the most important features are in certain PHATE coordinates)? The visualization plays a central role in this work, and I believe it should be clarified in more detail.

In the 2021 Cell Systems paper by the authors, all projections/visualization look similar across the different figures. However, here they all look different, even for the unperturbed cell cycle. Could the authors comment on why this is? I find that it greatly complicates interpreting the dynamics. How many dimensions is one using to project the dynamics on from the many original features? Do all unperturbed cases look different because the manifold is made in (say) 3D and the authors e.g. rotate it for their plots to highlight different aspects? Or has it something to do with the fact that different number of features are taken into account to start with: e.g. in the 2021 Cell Systems paper there were 48 features, and now there are 49 taken into account. Later in this work one also transitions to 37 features etc. These changes should also be more clearly explained/motivated.

In biology, transitions between different cell cycle states are often "irreversible" in the sense that they are controlled by sharp (potentially bistable) switches. Do the authors see signatures of this in their analysis and visualization? I do not see very clearly defined switching points/areas in the plots. Would it help to visualize the branch points in PHATE using the intrinsic dimension to visualize this?

3. Trajectories associated to different types of stress.

- Could the authors explain in more detail what happens over time in the presence of a certain stress? In the case of serum starvation, the manifold contracts in one direction over time (1d vs 7d), but it stays stretched out just as much in another dimension. How should we interpret this? Even after 7 days a subpopulation of cells are still in G2 with low p27, while many cells are in G0 with varying levels of higher p27. Are the G0 cells expected to further contract to a more well-defined location in the PHATE map over time? In the presence of etoposide, the "end state" is similarly stretched out. Are there other choices for the PHATE visualization where the long-term attracting state is more separated and well-defined?

- Related to the temporal component, cell cycle re-entry is not much discussed in this work, while it is mentioned in their Cell Systems paper. Could the authors comment on the probability, timing and molecular signatures of cells that re-enter the cell cycle?

- How does the strength of a certain stress affect the cell cycle arrest state and visualized manifold? Now it seems that each stress (serum starvation - etoposide - Palbociclib) corresponds to a different trajectory in the PHATE space. Is the type of stress indeed the main determinant of the trajectory, or could different strengths (concentrations) of a stressor lead to a continuum of trajectories? Also, to which extent does this affect the end state of the trajectory? A similar experiment using a different concentration could provide some new insights.

- I was confused when comparing Fig. 5B and Fig. EV5 as the trajectories of palbo and etop look very different from each other in Fig. EV5 and relatively similar in Fig. 5B. Why is this? I guess this relates to point 2 about how the visualization works and is chosen by the authors. It also shows that how we interpret the dynamics can greatly depend on such choices of visualization.

Reviewer #3:

Summary

In this study, the authors use a single cell imaging and manifold learning approach first described in Stallaert et al. 2021 to investigate cell cycle arrest. They treated RPE1 hTert cells with different stressors known to cause cell cycle arrest and used their approach to compare the molecular composition of these arrest states with asynchronously proliferating cells. They show that, as first described in Stalleart et al. 2021, cells are able to exit the cell cycle from both G1 and G2 in response to stress. They describe different states of G1-like arrest in response to hypomitogenic stress compared to oxidative or replicative stress. Finally, they show that both types of stress result in a convergence of 2C and 4C cells on a terminal or senescent state. Following replicative stress, they also report a polyploid population of cells resulting from re-entry into the cell cycle from this terminal state.

General remarks

Overall, the data are well presented and the conclusions well supported by the data. The authors have presented data providing a novel and detailed description of cell cycle exit in response to differing stressors. This work will be interesting for the cell cycle and proliferation, DNA damage response and senescence fields. However, many of the conclusions drawn are not particularly novel, and given that the analyses are limited to a single cell line, there remains a question about how relevant these results are across other cell lines. Testing their conclusions in at least one other non-cancer cell line (either by fixed or live cell imaging) would increase the impact of the work. There were also a few points in the manuscript where the data were difficult to interpret (Figs 4 and 5), but this could be improved by clearer labelling.

Major points

• Could the authors clarify how the manifold learning is applied to datasets with unknown states? Is it trained based on asynchronous cells then applied to other datasets or is it re-trained? If it is re-trained, could this introduce bias into characterising arrest states?

• In Fig 2H, data is plotted according to pseudotime, how do the numbers used correspond to the pseudotime cell cycle map plots and cell cycle progression? The data shown suggest waves of gene expression, does this represent cells cycling before arrest? The last timepoint shows an increase in p21 expression which is not reflected by an increase in CyclinD, as the expression of these genes mirrors each other at earlier pseudotimepoints and the expression of these proteins has been shown to correspond closely, do the authors think this is significant?

• Could the authors clarify how the 'terminal state' population Fig 4, is defined? There appears to be as much variation in gene expression within this group as there is between this population and the '8C state'.

• The authors use the p27K- reporter to verify their fixed cell imaging results by live cell imaging and then in the discussion state that p27 is controlled at the transcriptional level in response to stress. However, as far as I am aware, the p27K- reporter, as originally reported, has a constitutively active promoter, suggesting that, at least in the case reported here, a large level of p27 regulation is post-transcriptional. The authors should comment on this.

Minor points

• Labelling clarification: could the authors move the 'serum starvation' label in Fig 2A so that the data can be seen more clearly?

• Could the authors clarify if the data are plotted as 2D structures and if yes, include videos to show the 3D nature of the data as in Stalleart et al. 2021?

• Could the authors clarify where the arrest states are defined in Fig 3? They state that arrest states are 'characterized by a loss of RB phosphorylation' but this doesn't appear to be the case for the population closest to G2, are these cells arrested?

• Could the live cell imaging data of cells arresting in G1 vs G2 and measuring CDK2 activity and p21 levels from Fig 3F be plotted together? In this way the different fates of cells and the proportion of cells with these different fates will be clearer.

• Fig EV3A, could the authors use a darker grey colour as in Fig 3A to denote perturbed cells in the plots as in Fig 3? It is currently hard to distinguish them.

• In the 'senescence, mitotic skipping and polyploidy' section of the text, there is a typo: 'small proportion of cells also began to populate a region consisting entirely of polyploidy cells', 'polyploidy' should be 'polyploid'.

• In Fig 4A the relationships between the 'terminal state' 'mitotic skipping' and '8C' populations are unclear to me. Do cells with a

greater than 4C DNA content move from a terminal state to a 4C arrest state then follow the 8C trajectory?

• The figure legends for Fig 5 a and b are the wrong way round.

• Could the authors label the data shown in Fig5 and EV5 more to make clear the unperturbed and Palbociclib treated populations? The data and any differences between the Palbociclib and etoposide treated populations are currently quite hard to interpret.

• In Fig 5, could the authors show the data for Palbociclib at different timepoints? It would be interesting to see the evolution of the response to Palbociclib in these cells, as for the other treatments.

• In Fig 5, there appears to be a proportion of cells in etoposide treatment which arrest with a 4C DNA content but not from G2 but between the mitotic and G1 populations, can the authors comment on this?

• Is the proportion of cells which are polyploid in the 4i data comparable to that seen in the live cell imaging data in Fig 6D?

• Can the authors comment on what might determine whether cells in the terminal state re-enter the cell cycle to become polyploid. They describe how this is dependent on Cyclin D but if increased expression of Cyclin D (and other G1 markers) is a hallmark of the terminal state, why does this not result in cell cycle re-entry in more cells? This appears to be an interesting difference between replication and oxidative stress in that the latter does not result in polyploid cells and mitotic skipping.

• How do the authors define 'escape' from a terminally arrested state? If cells can re-enter S phase but do not divide, is this different from re-entry into a cell cycle ending in division? That is, to what extent do the authors think that mitotic skipping could contribute to an increase in tumour mass vs a full mitosis? Could this act as a starting point for genome amplification ahead of aneuploidy for example?

• Would the authors anticipate that Cyclin E overexpression would act like Cyclin D overexpression and push cells out of the senescence like arrest?

Reviewer #1:

In this paper the authors characterize the molecular architecture of cell cycle arrest induced using various stressors. By utilizing iterative immunofluorescence imaging the authors measure 49 cell cycle effectors in single RPE-1 cells in different cellular compartments and combine these measurements with measurements of cell morphology to generate a list of 2952 single cell features. They utilize dimensionality reduction to project this high-dimensional data into a 2D representation of a cell cycle 'map'. Using this map the authors identify points at which cells exit the cell cycle in response to different stresses and identify molecular mechanisms governing the decision to exit the cell cycle. This nicely builds upon the author's previous work which characterized the molecular structure of the mammalian cell cycle in unperturbed cells.

Overall the work presented by the authors is of a high standard, the data are believable and the conclusions are sound, and the paper will certainly be a useful resource for researchers in the field of cell biology, specifically related to cell cycle, single cell analysis, and DNA damage. However, I wanted to raise several concerns about the text which should be addressed prior to publication.

Main Points:

1. In Figure 1C the authors show measurements of phospho/total Rb projected onto the cell cycle map. They then define the proliferative trajectory and arrest trajectory based on cells being phospho-Rb positive and negative, respectively. However, it's not clear to me how the ratio of phospho/total Rb was used to identify populations of phospho-Rb negative and positive cells. I would recommend a clearer explanation of how these two populations were defined, i.e. was a threshold used? Could this threshold be indicated on the figure to aid the reader's understanding?

The reviewer is correct that we use a threshold value of phospho/total RB to distinguish arrested from proliferative cells. We have clarified this in the text and have included a figure showing this threshold (Fig EV1A).

Also in Figure 1C the levels of phospho/total Rb appear to be highest in G1 phase and then decline to an intermediate level as cells progress through S/G2/M phases. Given that Rb is hyperphosphorylated in G1 phase by CDK4/6 and CDK2 and that this phosphorylation is maintained through the cell cycle by these kinases, could the authors comment on why the level of Rb phosphorylation diminishes through S/G2/M phases?

Although there appears to be a subpopulation of cells in G1 with the highest phospho/total RB values (see the red cells at the bottom of the central arm of the structure), we do not find that phospho/total RB changes substantially across the proliferative phases (G1/S/G2/M), as show on the violin/boxplot below. The median phospho/total RB values (shown as the white dot in the boxplot) are the same across all proliferative phases.

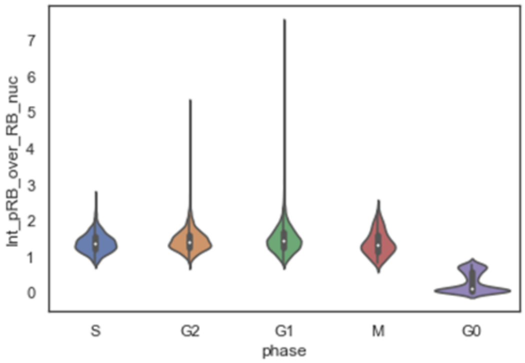

2. The authors claim that: "Trajectory inference by diffusion pseudotime revealed that serum-starved cells diverge from the proliferative cell cycle during G2 (Fig 2B)." I find this statement a little confusing. Would it be possible to provide a more detailed explanation of this method and how the authors used it to arrive at this conclusion?

We now provide a more detailed description of diffusion pseudotime earlier in the manuscript and provide the reference to the original work describing the method (lines 122-125).

3. In Figure 2A-B the authors annotate the cell cycle trajectory map indicating G2 phase cells occupy the top right corner, with mitotic cells indicated to the left of these G2 cells. In subsequent panels, Figure 2D-E,I the authors depict G2 phase cells in a different location on the map, appearing in the location where mitotic cells are defined in Figure 2A-B. The Figure legend provides no description of these annotations. A clearer description of how these populations of cells are defined, and why the location of G2 cells specifically appears to change in the different figure panels would be helpful.

We have relabeled this figure more clearly and have included a description of how the proliferative phases are annotated earlier in the manuscript (lines 121-122, see Fig 1B).

4. The authors state "While unperturbed cells exhibited a clear increase in cyclin D1 during G2, serum starvation significantly reduced the induction of cyclin D1 during G2, leading to less inherited cyclin D1 in daughters cells (Fig 2G) (Guo et al, 2005)." It is clear that serum starvation reduced the levels of Cyclin D in both mother and daughter cells during G2 phase compared to control cells. However, no statistical test is provided to demonstrate that this difference is indeed significant. There is no description of what the solid line and shaded region represent in this figure panel. Could a statistical test be performed to qualify the statement that the difference is indeed significant? Perhaps by calculating a fold-change in Cyclin D levels during G2 phase in control and serum starved cells?

We thank the reviewer for identifying this oversight. We have indicated in the figure 2 legend that the solid lines represent population medians and the shaded areas are the 95% confidence intervals. In effect, these shaded areas provide a visualization of the statistical differences along these curves.

In addition, previous work (PMID: 28869970) has identified that loss of mitogen signalling in the mother cell leads to a reduction in Cyclin D mRNA which is inherited by daughter cells, leading to lower levels of Cyclin D in daughters. Therefore, the reduced levels of Cyclin D observed in daughter cells after serum starvation could be due to a combined effect of downregulated Cyclin D protein in mother cells, which is inherited by daughter cells, as well as less Cyclin D mRNA inherited by daughter cells.

We have added a sentence (lines 158-160) in the Results citing this work and its relation to the current study.

5. There are several places in the text where the authors describe a piece of data from a figure panel followed by a reference to another paper. These references seem out of context to me, since the text preceding these references only describes results from the current paper and makes no link to the work described in the cited papers. Here are a few examples:
a. "In the unperturbed cell cycle, cyclin D1 increased in late G2 and remained elevated during mitosis and after cell division (Fig 2D, left panel) (Gookin et al, 2017)."
b. "While unperturbed cells exhibited a clear increase in cyclin D1 during G2, serum starvation significantly reduced the induction of cyclin D1 during G2, leading to less inherited cyclin D1 in daughters cells (Fig 2G) (Guo et al, 2005)."
c. "...we confirmed that p27 increased steadily in individual cells following serum starvation, starting immediately after cell division (Fig 2J) (Coats et al, 1996)."

It's not clear to me what point the authors are making that are meant to be supported by these references. I assume it's because these other papers have shown similar results. If that's the case, I would recommend making that clearer by adding to the text a statement like "....as shown previously (reference)."

In general, more care could be taken throughout the manuscript to more explicitly state whether the findings in this manuscript are similar to or contradictory to previous results and to credit these previous studies appropriately.

We thank the reviewer for pointing out this oversight. We have amended the text to more explicitly state how the results in the current study relate to those in previous studies.

6. In Figure 2H and I, the authors find that p27 levels are elevated in serum starved cells. The authors then state they used the mVenus-p27K- biosensor to confirm that p27 levels steadily rise after serum starvation in Figure 2J. However, the data presented in Figure 2J cannot be used to support that conclusion. The mVenus-p27K- biosensor was not designed to reflect endogenous p27 levels, but rather was originally designed as a qualitative marker of G0. Its expression is under the control of a constitutive promoter and that is the primary reason why the authors find its levels increasing linearly after the mitosis following serum starvation. It does not reflect the transcriptional control of endogenous p27 as implied by the authors. In fact, in the Discussion section, the authors even state that p27 is primarily regulated through its 5'UTR of the mRNA, which would clearly be lacking in the mVenus-p27K- biosensor. I would recommend they remove this figure. Their data in Figure 2H and I can be used to support their statement that p27 levels were higher in serum starved cells and its levels increased with pseudotime.

We thank the reviewer for pointing this out. We have removed the figure.

7. The area of the map in Figure 4A that is labeled Terminal State appears to be labeled with Endoreduplication State in Figure 6A. These two states seem like they would be mutually exclusive, since one term implies a lack of cycling and the other term implies cells are cycling (just not dividing). A clearer reasoning behind the annotation of these two maps is needed. Its not clear why these two states clustered together when I would think they would be quite different states. The authors suggest that perhaps a small fraction of cells is escaping the Terminal State to begin endoreduplicating. However the number of cells in the endoreduplication arm appears to be much greater than the <5% of cells that were observed to escape the Terminal Phase (Fig 6D). An alternative explanation could also be that these are simply all Endoreduplicating cells. Perhaps the authors could include some single-cell traces of CDK2 activity and p21 expression for Terminally State cells after Etoposide treatment to better support these conclusions. I would expect terminal state cells to have low CDK2 activity and high p21 levels until at least 4 days after Etoposide treatment.

We agree with the reviewer that the previous labeling was confusing. The terminal state in Figure 4 refers to that entire region that we later show to be senescent cells. We have relabeled this region "senescence" for consistency and clarity. We show that on rare occasions (<5%), cells can escape from this state (this area of the map) and undergo endoreduplication to generate the 8C cells that we also observe on the upper right area of the map. However, as the reviewer states, the cells in this terminal/senescent state are not actively cycling. In Fig 6, we meant to imply that endoreduplication occurs when

“escape” from this region, and the arrow was meant to indicate this fate (i.e. that these cells leave this region). We have clarified the labeling of these maps.

8. The authors state "Thus, regardless of the phase of cell cycle exit (and DNA content), the senescent state resembles a G1-like molecular state, which narrows the mechanisms that stabilize this cell cycle arrest-as well as those that could reverse it-to G1 regulatory events". The authors support this statement by trying only 2 things to reverse the senescent state: over-expressing Cyclin D or Cyclin A2. This is far from an extensive test of possible reverse mechanisms. Thus, I would recommend the authors tone down this claim and amend the text such as "....which potentially narrows the mechanisms that stabilize this cell cycle arrest....", or something similar.

We agree with the reviewer and have tempered the language as suggested.

Minor Points:
1. In figure 2G, its not clear when the cells were serum starved relative to the data on the axis. It would be helpful to include that information in the figure legend.

We have updated the legend to indicate that we serum starved cells for at least 8 h prior to imaging.

2. The authors use terms such as "G0-Like state", "G2-like state" (section on Replication stress), "pseudo-G1 molecular state" (section on senescence), and "terminal state". None of these states are very well defined in the text. I would recommend more clearly defining what is meant by each term and how they are different from each other. In the case of G0-like vs Pseudo-G1, it sounds like the terms could be interchangeable. In which case, it would probably more clear for the reader to choose one term. If they are not interchangeable, then it should be stated more clearly what the difference between these two states are.

We agree and thank this reviewer for this suggestion. We have simplified our nomenclature throughout the text, replacing “terminal state” with “senescence” and using “G1-like” alone to describe the state after mitotic skipping.

3. In the text, the authors state they stained for 49 cell cycle proteins. However, in figure 1A, the schematic says they stained 50 proteins. Furthermore, in the Extended Data Table EV1, there are only 45 proteins listed along with 2 morphological parameters. In Figure 2H and Figure EV2F,G, the heatmaps includes 47 proteins. Thus, there is an inconsistency throughout the manuscript and figures about how many proteins were actually stained for in the 4i experiments. I would recommend the authors recheck the manuscript to make it more clear how many and which proteins were stained for in each experiment.

We apologize to the reviewer for this oversight. We stained for 49 proteins (+DNA), however, two of the antibodies were excluded due to poor staining across the samples.

We have changed the schematic to 47 proteins and removed these two antibodies from Table EV1. There indeed are 47 features listed in Dataset EV1 out of 2952 total features measured in the entire experiment, but this was a coincidence. These are a list of “features” used as inputs to PHATE, which were selected based on a principled feature selection performed in our previous study (Stallaert et al, Cell Systems 2022), and not a list of the total antibodies that were used.

4. The authors state: "Over several days of etoposide treatment cells proceed further along the 2C and 4C arrest trajectories, transitioning through additional molecular states (Fig 3A inset), which we will discuss in greater detail below." When looking at the inset in Fig3A it's not clear what the "additional molecular states" are that the cells transition through. Perhaps some annotation to highlight these "additional molecular states" would be useful to aid the reader's interpretation of the data.

We have clarified in the text that we are referring to the states in the upper portions of the map that were not yet discussed.

5. There is a typo in results section when referring to loss of CDH1 and SKP2 during senescence. The figure reference is to "(Fig N-O)". Presumably this should be "(Fig 4N-O)"

We have corrected this.

Reviewer #2:

In this manuscript, Stallaert and coworkers have used hyperplexed imaging of 49 cell cycle effectors and manifold learning to obtain a map of the molecular architecture that governs cell cycle exit and progression into reversible ("quiescent") and irreversible ("senescent") arrest states. They found different paths of cell cycle arrest, specific to the types of applied stress.

This work extends recently published work by the authors (The structure of the human cell cycle - Cell Systems - 2021) by visualizing and interpreting in more detail the ways cells can go into cell cycle arrest in response to different stress signals. The manuscript is well written and interesting to read, clearly visualizing different trajectories leading to cell cycle arrest, including the molecular signatures along those trajectories.

Overall, the analyses and presented results deserve to be published somewhere. However, I remain on the fence about whether the findings fulfill the requirement of "conceptual novelty" required for publication in Molecular Systems Biology. In their recent Cell Systems paper, the authors have already presented the methodology and visualization method of the cell cycle, characterized the mechanisms driving cell cycle exit and re-entry, analyzed molecular signatures of cell cycle arrest and senescence, and even showed different cell cycle arrest trajectories (towards senescence) from both G1 and G2. Therefore, the last sentence of the abstract claiming that this work "provides a first glimpse of the overall organization of cell

proliferation and arrest" seems to be overselling the manuscript as much has already been published in their 2021 Cell Systems paper.

We thank the reviewer for these comments. In the previous manuscript, we presented our cell cycle mapping approach and used it to plot the unperturbed cell cycle. In the current manuscript, we are asking a different question: how does the cell cycle respond to different stresses? By treating cells with three different stresses, we reveal multiple, distinct states of cell cycle arrest, most of which were not detected in our previous work. Furthermore, because of the high-dimensional signature we obtained for each cell, we can demonstrate how these arrest states differ from one another at a detailed molecular level. We also describe additional components of cell cycle arrest not explored in the first paper, including mitotic skipping, escape from senescence and endoreduplication to generate polyploidy. By plotting all of these trajectories of cell cycle arrest on a single map, we believe that we do present "a first glimpse of the overall organization of cell proliferation and arrest". We have endeavored to clarify the novelty of this work throughout the manuscript.

Apart from this main concern of conceptual novelty, here below several other questions that need to be addressed, likely possible by re-analyzing existing data, rewriting parts of the paper, and/or carrying out a few similar additional experiments.

1. Machine learning.

One common criticism of some machine-learning approaches is that they are often used in recent times to re-discover known biology. Such confirmation is in fact already interesting. Ideally though, machine-learning approaches also uncover some new biology and suggest future experiments to test these findings/suggestions. The authors already discuss both aspects, but I think it could be more clearly delineated which findings are new vs. what was known already, and how their new findings trigger new experiments/questions.

A similar point was raised by reviewer #1, and we have addressed this point in several places throughout the text (e.g. lines 135-137, 158-160, 167-170, etc).

I also believe it would be good to spend some time to explain in basic terms what "manifold learning" and the PHATE visualization are. By (partially) de-mystifying such machine-learning approaches, I think readers will be able to better understand the results. Moreover, while the visualization is nice to see qualitative differences in the sense that different stresses lead to different trajectories, I wonder whether the authors could go a bit further in interpreting/explaining these denoised lower-dimensional embeddings (see next comment)?

We have added additional text to help demystify manifold learning and what it can reveal in the current manuscript.

2. PHATE visualization and interpretation.

How should we interpret the coordinates in the lower dimensional representation using PHATE? In a typical PCA, the coordinates are just a linear combination of the original coordinates, allowing to directly interpret the meaning of each principal component. Can we do something similar in PHATE? If so, could you use this to clarify your plots and learn something new (e.g. indicating what the most important features are in certain PHATE coordinates)? The visualization plays a central role in this work, and I believe it should be clarified in more detail.

PHATE differs from PCA in that it is a non-linear dimensionality reduction technique that uses diffusion probabilities to encode local and global similarities based upon the expression of features amongst cells and then squishes those into two dimensions based on stress strain optimization. As such, the lower dimensional dimensions of PHATE embeddings are not interpretable in the same way that PCA coordinates are.

In the 2021 Cell Systems paper by the authors, all projections/visualization look similar across the different figures. However, here they all look different, even for the unperturbed cell cycle. Could the authors comment on why this is? I find that it greatly complicates interpreting the dynamics. How many dimensions is one using to project the dynamics on from the many original features? Do all unperturbed cases look different because the manifold is made in (say) 3D and the authors e.g. rotate it for their plots to highlight different aspects? Or has it something to do with the fact that different number of features are taken into account to start with: e.g. in the 2021 Cell Systems paper there were 48 features, and now there are 49 taken into account. Later in this work one also transitions to 37 features etc. These changes should also be more clearly explained/motivated.

We thank the reviewer for pointing out this ambiguity. First, in the original Cell Systems paper we used 48 antibodies to generate the structure; here we used 47 antibodies, except for the senescence 4i experiment where we used 37. Each antibody used (plus one for DNA/Hoechst) corresponds to a distinct image that we obtained in each experiment. The antibodies were chosen specifically for the question we wanted to address (i.e., for the map we wanted to obtain). In the above mentioned cases, we chose antibodies against known cell cycle effectors when we wanted to visualize the cell cycle, and when we wanted to visualize the path to senescence, we chose a (mostly) different set of antibodies that reflected known changes that occur in senescence. From these images, we derive many more additional "features", including for example the abundance of a given protein in different cellular compartments, morphological features such as size and shape of the nucleus and derivative features such as cytoplasmic/nuclear and phospho/total protein ratios. We have clarified this in the text (lines 101-105).

The reason that the maps look different in different figures is that they are different embeddings (projections). Although distances between individual cells are meaningful, some aspects of the overall shape of the structure are less important. For example, the structure can be rotated, reflected, or slightly stretched without altering the

interpretation. The precise structure shape rendered for each data set often varies depending on which subset of data is used to produce the structure. For example, in Fig 1 we performed manifold learning on *just* the unperturbed cells and obtained the map that is shown. In Fig 2, we performed manifold learning on the combined dataset of unperturbed *and* serum-starved cells. Because PHATE captures both local and global manifold structure, the inclusion of serum-starved cells (and their distinct molecular signatures) changes the projection of the unperturbed cell cycle, effectively squeezing it horizontally towards the right side of the projection to accommodate the structure of the hypomitogenic arrest trajectory on the left side. The spatial relationships between cells of the unperturbed structure are mostly retained and we still can see the same topography (a cyclical proliferative trajectory and linear arrest trajectory). We have clarified in the text when the structures shown are new embeddings to avoid this confusion.

In biology, transitions between different cell cycle states are often "irreversible" in the sense that they are controlled by sharp (potentially bistable) switches. Do the authors see signatures of this in their analysis and visualization? I do not see very clearly defined switching points/areas in the plots. Would it help to visualize the branch points in PHATE using the intrinsic dimension to visualize this?

This is an excellent point. In fact, we do see evidence of rapid switching between molecular states in the cell cycle structure visualization. The best examples are the transition from G1 to S and the transition from M to G1/G0. In the structure shown in Fig 1B, both transitions feature a sparse distribution of cells flanked by dense distributions on either side. This pattern indicates a rapid transition through an intermediate molecular state. What is not discernible from these plots, however, is the directionality of the transition (i.e., G1 to S as opposed to S to G1). Directionality must be inferred from historical knowledge or from a time series of cell cycle structures, as shown in Fig 4B. In future studies, we intend to develop a modeling framework that will infer switch-like transitions directly from the 4i data, using probabilistic models to simulate the behavior of individual cells along the points in the structure.

3. Trajectories associated to different types of stress.

- Could the authors explain in more detail what happens over time in the presence of a certain stress? In the case of serum starvation, the manifold contracts in one direction over time (1d vs 7d), but it stays stretched out just as much in another dimension. How should we interpret this? Even after 7 days a subpopulation of cells are still in G2 with low p27, while many cells are in G0 with varying levels of higher p27. Are the G0 cells expected to further contract to a more well-defined location in the PHATE map over time? In the presence of etoposide, the "end state" is similarly stretched out. Are there other choices for the PHATE visualization where the long-term attracting state is more separated and well-defined?

Yes, it appears that after 7d of starvation, cells occupy a smaller space of the map than after 1d. We do not observe any significant differences in protein expression/activity between these conditions, so we cannot propose with confidence what this difference means. Our hypothesis is that the space on the map uniquely occupied by cells in the 1d condition may represent cells re-entering the cell cycle from this state of arrest. These cells occupy a space between the hypomitogenic G0 and G1 of the proliferative cell cycle and start to possess higher levels of RB phosphorylation as they move towards G1, consistent with cell cycle re-entry. However, we have no direct evidence to support this claim so we did not include it in the manuscript.

As the reviewer notes, in the case of both starvation and etoposide, we observed less heterogeneity over time, as cells converge on a smaller and smaller region of the overall map.

- Related to the temporal component, cell cycle re-entry is not much discussed in this work, while it is mentioned in their Cell Systems paper. Could the authors comment on the probability, timing and molecular signatures of cells that re-enter the cell cycle?

In the current manuscript, we do not have sufficient data to comment on cell cycle re-entry (other than the endoreduplication we observe after mitotic skipping, which we observed directly by time-lapse imaging). Cell cycle re-entry from these other arrest states is indeed an interesting question and we are currently pursuing this topic in another project.

- How does the strength of a certain stress affect the cell cycle arrest state and visualized manifold? Now it seems that each stress (serum starvation - etoposide - Palbociclib) corresponds to a different trajectory in the PHATE space. Is the type of stress indeed the main determinant of the trajectory, or could different strengths (concentrations) of a stressor lead to a continuum of trajectories? Also, to which extent does this affect the end state of the trajectory? A similar experiment using a different concentration could provide some new insights.

Each stress (serum starvation/etoposide/palbociclib) induces a categorically distinct molecular mechanism to induce arrest (i.e. loss of cyclin D, p21 induction and inhibition of CDK4/6, respectively). Therefore, we do not expect that changes in the quantity of the stress will induce qualitatively different arrest responses, but rather different proportions of cell cycle arrest among the population (see Figure below).

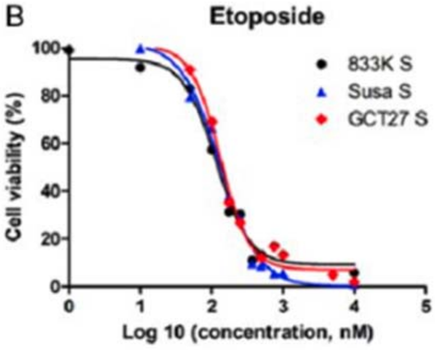

Fig 1. Etoposide dose-response curve. From Yeste-Velasco et al. Am J Cancer Res (2019)

- I was confused when comparing Fig. 5B and Fig. EV5 as the trajectories of palbo and etop look very different from each other in Fig. EV5 and relatively similar in Fig. 5B. Why is this? I guess this relates to point 2 about how the visualization works and is chosen by the authors. It also shows that how we interpret the dynamics can greatly depend on such choices of visualization.

In Fig EV5, we treated with palbo for 24h while in Fig 5B we treated for 4 and 8 days. We also measured a very different set of proteins for the 4i experiment in Fig 5B because we wanted to specifically resolve the molecular trajectory into senescence. These data show that 24h of palbo treatment induces a distinct arrest trajectory (compared to all other stresses, including etoposide), but after 4-8 days, cells converge on a similar senescent state as observed following sustained etoposide treatment (4-8d). These data are used to show that, despite the phase and mechanism of cell cycle exit, cells converge on a similar state of senescence with a G1-like molecular profile. We outline the difference between these experiments/figures in the text and figure legends.

Reviewer #3:

Summary

In this study, the authors use a single cell imaging and manifold learning approach first described in Stallaert et al. 2021 to investigate cell cycle arrest. They treated RPE1 hTert cells with different stressors known to cause cell cycle arrest and used their approach to compare the molecular composition of these arrest states with asynchronously proliferating cells. They show that, as first described in Stalleart et al. 2021, cells are able to exit the cell cycle from both G1 and G2 in response to stress. They describe different states of G1-like arrest in response to hypomitogenic stress compared to oxidative or replicative stress. Finally, they show that both types of stress result in a convergence of 2C and 4C cells on a terminal or senescent state. Following replicative stress, they also report a polyploid population of cells resulting from re-entry into the cell cycle from this terminal state.

General remarks

Overall, the data are well presented and the conclusions well supported by the data. The authors have presented data providing a novel and detailed description of cell cycle exit in response to differing stressors. This work will be interesting for the cell cycle and proliferation, DNA damage response and senescence fields. However, many of the conclusions drawn are not particularly novel, and given that the analyses are limited to a single cell line, there remains a question about how relevant these results are across other cell lines. Testing their conclusions in at least one other non-cancer cell line (either by fixed or live cell imaging) would increase the impact of the work. There were also a few points in the manuscript where the data were difficult to interpret (Figs 4 and 5), but this could be improved by clearer labelling.

We thank the reviewer for their helpful suggestions. We have included data using an additional, non-transformed cell line (MCF10A breast epithelial cells), validating the specific observations that: (1) cells may exit from G1 or G2 following replicative stress (Fig EV2H); (2) cells undergo mitotic skipping following G2 exit (EV4E-H); (3) through mitotic skipping, cells transition into a G1-like, SA-beta-galactosidase-positive senescent state (EV4E-H); and (4) cells may re-enter the cell cycle from this senescent state and undergo endoreduplication to generate 8C polyploid cells (EV4M).

Major points

• Could the authors clarify how the manifold learning is applied to datasets with unknown states? Is it trained based on asynchronous cells then applied to other datasets or is it re-trained? If it is re-trained, could this introduce bias into characterising arrest states?

Manifold learning does not involve training in the same way that other machine learning models do (e.g. random forest, neural networks). It is simply a non-linear dimensionality reduction technique that preserves both local and global structure. It takes as input a dataset consisting of many cells with many measurements per cell, determines which cells are similar to each other according to this multi-feature signature, and then places them close to one another in lower dimensional space. The final result is a low-dimensional map (often two or three dimensions). We have added a more detailed description of manifold learning and what it does in the Results (lines 105-115) to help clarify the approach. We performed manifold learning on each dataset to produce a map consisting of all the cells in that dataset. For example, in Fig 1, we make a map of just the unperturbed cells. In Fig 2, on the other hand, we make a map using the combined dataset of unperturbed and serum-starved cells. This preserves the overall structure of the unperturbed cell cycle but "repositions" them relative to the hypomitogenic arrest state where the serum-starved cells reside. A similar repositioning happens when we include etoposide-treated cells with unperturbed cells in Fig 3. Fig 7 projects all of the arrest states and the proliferative cell cycle into a single manifold. However, in each case, both the local and global relationships among the cells in each state are preserved. Therefore, manifold learning using PHATE preserves trajectories/states, even if additional states are present in the embedding.

• In Fig 2H, data is plot ed according to pseudotime, how do the numbers used correspond to the pseudotime cell cycle map plots and cell cycle progression? The data shown suggest waves of gene expression, does this represent cells cycling before arrest? The last timepoint shows an increase in p21 expression which is not reflected by an increase in CyclinD, as the expression of these genes mirrors each other at earlier pseudotimepoints and the expression of these proteins has been shown to correspond closely, do the authors think this is significant?

Pseudotime in Fig 2H is derived from the same pseudotime measurements shown in Fig 2B (right panel). Each cell in Fig 2B possesses a pseudotime va For Fig 2H we simply bin cells over pseudotime and display the mean intensity of each feature in each pseudotime bin. We have changed the scale in Fig 2H to be consistent with Fig 2B.

We mention in the Results that p21 (along with p27) is one of the proteins that is not completely downregulated along the hypomitogenic arrest trajectory. It is indeed possible that this increase in p21, along with additional increases in p53 and phospho-CHK1 also observed, does occur as cells transition into deep states of hypomitogenic arrest. This signature would be consistent with a DNA damage response and we may explore this possibility in future experiments.

• Could the authors clarify how the 'terminal state' population Fig 4, is defined? There appears to be as much variation in gene expression within this group as there is between this population and the '8C state'.

We have relabeled this terminal state as senescence for clarity and consistency in the paper. Several computational and experimental approaches distinguish this state from the 8C state. First, PHATE will place cells in this lower dimensional space based on their similarity in gene expression (or in our case protein abundance/activity). The separation of cells into these two regions on the map is determined by their relative differences in molecular signature. Second, if we perform graph-based clustering using the Leiden algorithm to cluster cells by their molecular signatures, we also distinguish the senescent cells from the 8C cells (see below). Finally, these groups differ in ways that are biologically meaningful. The 8C state contains polyploid cells with 8 copies of DNA. We also detect clear differences in expression of most senescent markers between these two groups (Fig 4D-K).

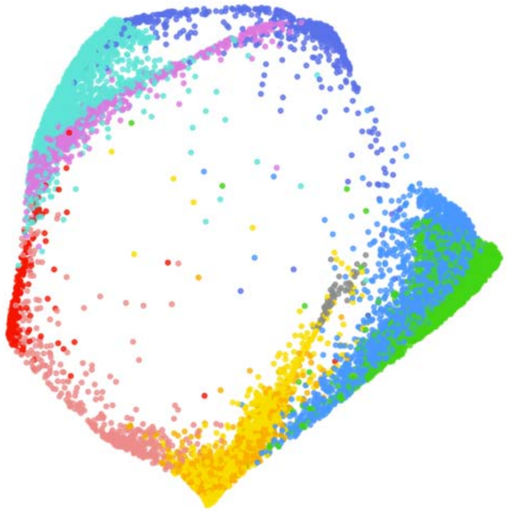

Fig 2. Leiden clustering

• The authors use the p27K- reporter to verify their fixed cell imaging results by live cell imaging and then in the discussion state that p27 is controlled at the transcriptional level in response to stress. However, as far as I am aware, the p27K- reporter, as originally reported, has a constitutively active promoter, suggesting that, at least in the case reported here, a large level of p27 regulation is post-transcriptional. The authors should comment on this.

Reviewer #1 made a similar comment. We agree with both reviewers and have removed the figure.

Minor points

• Labelling clarification: could the authors move the 'serum starvation' label in Fig 2A so that the data can be seen more clearly?

We have updated the figure.

• Could the authors clarify if the data are plotted as 2D structures and if yes, include videos to show the 3D nature of the data as in Stalleart et al. 2021?

All the data presented are 2D projections.

• Could the authors clarify where the arrest states are defined in Fig 3? They state that arrest states are 'characterized by a loss of RB phosphorylation' but this doesn't appear to be the case for the population closest to G2, are these cells arrested?

Indeed, we show that the trajectory of etoposide-treated cells diverged from the unperturbed cell cycle during G2 and we see the abrupt transition of these cells from a proliferative G2 state to an arrested state that occurs as the cells undergo mitotic skipping and lose expression of cyclin A (Fig 4L), after which cells remain arrested along the rest of the arrest trajectory.

• Could the live cell imaging data of cells arresting in G1 vs G2 and measuring CDK2 activity and p21 levels from Fig 3F be plotted together? In this way the different fates of cells and the proportion of cells with these different fates will be clearer.

It would only be possible to plot the G2 phases of the mother cells that undergo mitosis (on the left) with the G2 phase of arrested cells (on the right). However, the time scales of these two groups are very different. Cells that go to mitosis do so in 5-10 hours, while the arrested cells remain so for the length of the experiment (>80h). Because of this, it would be very difficult to present both on the same graph for comparison. We have made sure to keep the scales close to allow easy comparison of the signals across graphs.

• Fig EV3A, could the authors use a darker grey colour as in Fig 3A to denote perturbed cells in the plots as in Fig 3? It is currently hard to distinguish them.

We have updated the figure.

• In the 'senescence, mitotic skipping and polyploidy' section of the text, there is a typo: 'small proportion of cells also began to populate a region consisting entirely of polyploidy cells', 'polyploidy' should be 'polyploid'.

We thank the reviewer for spotting this typo. We have corrected it in the revised manuscript.

• In Fig 4A the relationships between the 'terminal state' 'mitotic skipping' and '8C' populations are unclear to me. Do cells with a greater than 4C DNA content move from a terminal state to a 4C arrest state then follow the 8C trajectory?

We have updated our labeling (as suggested by reviewer #1 as well) to provide more clarity and consistency across the manuscript.

• The figure legends for Fig 5 a and b are the wrong way round.

We have updated this legend.

• Could the authors label the data shown in Fig5 and EV5 more to make clear the unperturbed and Palbociclib treated populations? The data and any differences between the Palbociclib and etoposide treated populations are currently quite hard to interpret.

These plots are meant to show that, despite diverging from the proliferative cell cycle at different phases in response to different mechanisms, cells converge on a similar state of senescence following palbo or etop treatment. The fact that PHATE places cells from both conditions in the same "terminal state" (at the top of the leftmost region) is evidence that their molecular signatures are highly similar. We have added a label to highlight this state to Fig 5 and Fig EV5

• In Fig 5, could the authors show the data for Palbociclib at different timepoints? It would be interesting to see the evolution of the response to Palbociclib in these cells, as for the other treatments.

We also were interested in how these states evolve but we observed no meaningful difference in states of arrest from 4 to 8 days for both etop- and palbo-treated cells (see below).

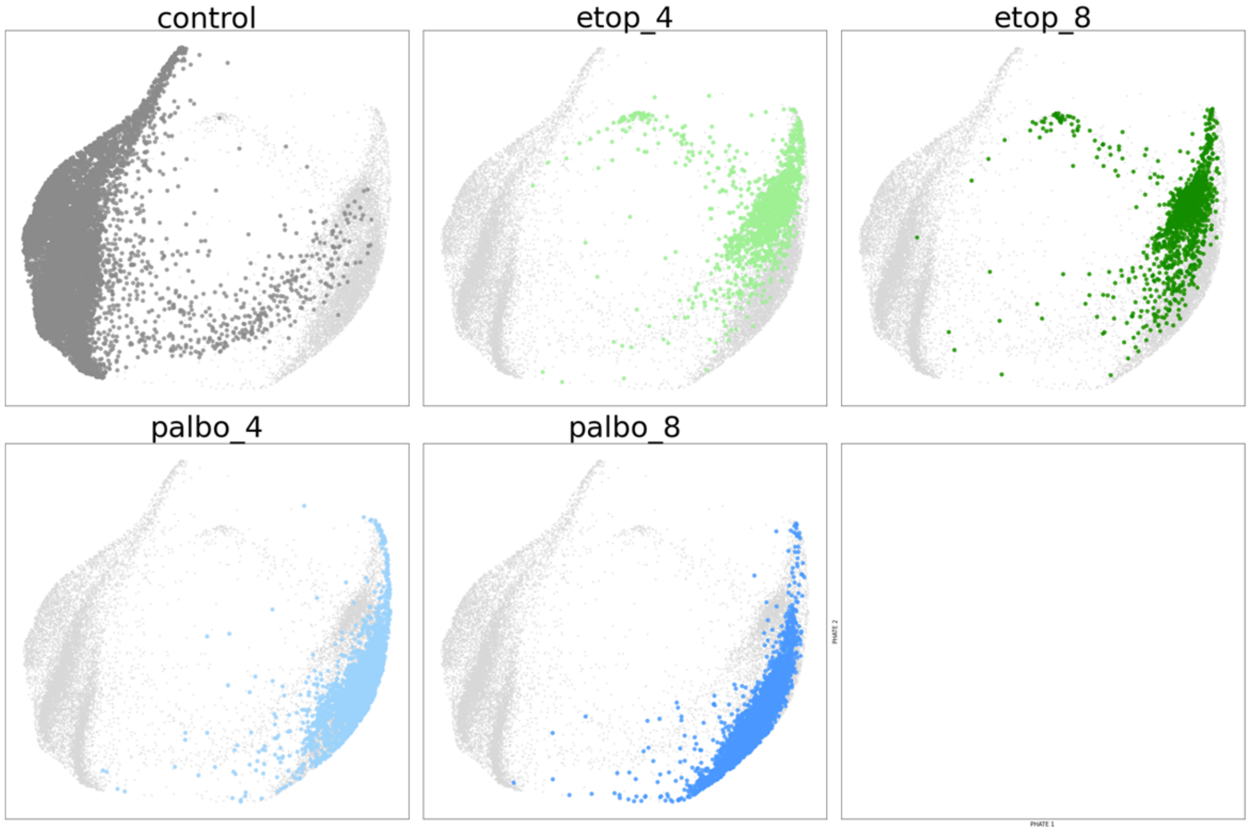

Fig 3

• In Fig 5, there appears to be a proportion of cells in etoposide treatment which arrest with a 4C DNA content but not from G2 but between the mitotic and G1 populations, can the authors comment on this?

We appreciate the keen eye of the reviewer. It is possible that these are cells that tried to re-enter the cell cycle following mitotic skipping but arrested prior to S phase.

• Is the proportion of cells which are polyploid in the 4i data comparable to that seen in the live cell imaging data in Fig 6D?

We thank the reviewer for the idea to calculate this. We also find ~5% of etoposide-treated cells are polyploid after 4 days of treatment (the same length as the time-lapse experiment). We have added this result to the manuscript.

• Can the authors comment on what might determine whether cells in the terminal state re-enter the cell cycle to become polyploid. They describe how this is dependent on Cyclin D but if increased expression of Cyclin D (and other G1 markers) is a hallmark of the terminal state, why does this not result in cell cycle re-entry in more cells? This appears to be an interesting

difference between replication and oxidative stress in that the latter does not result in polyploid cells and mitotic skipping.

Indeed, increasing cyclin D to supraphysiological levels can push cells out of this senescent state. However, this fate decision is almost certainly dependent on other factors in addition to cyclin D. Control of CDK activity is known to be regulated in a stoichiometric manner by competition between cyclins and CDK inhibitors such as p21 and p27 (PMID: 28869970). The capacity of a cell to escape from this arrest likely emerges from the combined expression and activity of a number of cell cycle effectors, thus making escape, even in the presence of high cyclin D, a low probability event. We agree with the reviewer that this may be an interesting difference between replicative and oxidative stress, however, as we state in the manuscript, we believe that the effect of H2O2 is merely more transient than etoposide, increasing the probability that cells will repair any DNA damage and re-enter the cell cycle before entering a senescent state (see EV3H-I).

• How do the authors define 'escape' from a terminally arrested state? If cells can re-enter S phase but do not divide, is this different from re-entry into a cell cycle ending in division? That is, to what extent do the authors think that mitotic skipping could contribute to an increase in tumour mass vs a full mitosis? Could this act as a starting point for genome amplification ahead of aneuploidy for example?

We agree with the reviewer that this is an interesting phenomenon. In our experimental conditions, it is true that cells arrest following endoreduplication. However, in these conditions, etoposide remains present and so these cells likely accumulate new DNA damage during endoreduplication and arrest in their subsequent G2, prior to cell division. We do not know if these cells would continue through to mitosis if etoposide were not present, to generate a cycling tetraploid population. Whole genome duplication can facilitate tumorigenesis and is becoming an area of interest for cancer therapy (Fujiwara et al. Nature 2005, Quinton et al. *Nature* 2021; see links below). It would be interesting to examine the role of mitotic skipping and endoreduplication in the context of cancer initiation and progression. We thank the reviewer for this suggestion and have added additional text to the Discussion.

https://www.nature.com/articles/nature04217
https://www.nature.com/articles/s41586-020-03133-3

• Would the authors anticipate that Cyclin E overexpression would act like Cyclin D overexpression and push cells out of the senescence like arrest?

We do expect that increased expression cyclin E, another G1 cyclin, may also be able to push cells out of this senescent state. Increased Cyclin E is capable of inducing cell cycle reentry from other stably arrested G1 states, such as those induced by CDK4/6 inhibitors (https://doi.org/10.1016/j.tcb.2021.05.001)

RE: MSB-2022-11087R, The molecular architecture of cell cycle arrest

Thank you for sending us your revised manuscript. We have now heard back from the two reviewers who were asked to evaluate your revised study. Both reviewers think that the study has improved as a result of the performed revisions. While reviewer #3 is supportive of publication, reviewer #2 lists some remaining concerns, which we would ask you to address in a last round of revision. These concerns can be addressed by text modifications and addition of further discussion clearly describing how the study advances our understanding of the cell cycle.

Before we formally accept the manuscript for publication we would ask you to address some remaining editorial issues listed below.

Reviewer #2:

I appreciate that the authors have introduced a clearer and more elaborate explanation of the manifold learning / PHATE embedding used in the manuscript. It was also useful to clarify that the different maps look different in the figures because they correspond to different embeddings. Apart from this, it is not entirely clear to me whether other comments have led to additional changes to the manuscript, as there was no list of changes to the manuscript provided.

While many of the comments and questions were addressed in a reasonably satisfactory way, and the work is very good and worth publishing, my main concern of (lack of) conceptual novelty (a requirement for publication in Molecular Systems Biology) remains. The authors addressed this point by repeating that this work extends their previous Cell Systems (2021) paper by studying the effect of three different stresses. In my opinion, this is not a particularly convincing argument without clearly showing corresponding new findings and conclusions. I repeat some of my previous comments related to this critical point, which I believe should be better addressed.

1. Comment 1 previously stated: " ... The authors already discuss both aspects, but I think it could be more clearly delineated which findings are new vs. what was known already, and how their new findings trigger new experiments/questions."

In response, the authors say. "We have addressed this point in several places throughout the text (e.g. lines 135-137, 158-160, 167-170, etc)." However, in all these lines, the authors discuss findings that were known already, citing the relevant works. This is perfectly fine of course, but it does not address which of their findings is new and how it triggers new experiments/questions. While I think the visualization using the PHATE embedding is beautiful, it would be good to see more clearly how it increases our understanding of the cell cycle.

2. Despite my objection, the last sentence of the abstract still claims that this work "provides a first glimpse of the overall organization of cell proliferation and arrest", which I argued oversells the manuscript.

The brief abstract of their 2021 Cell Systems paper reads "A complete visualization of the human cell cycle is rendered by combining highly multiplexed single-cell imaging and manifold learning. The molecular trajectories through the proliferative and arrested cell cycle are revealed, and the mechanisms governing cell cycle exit and re-entry are characterized. In addition, the multivariate molecular signature that defines cellular senescence is identified."

The authors thus literally mention themselves in their previous work that they reveal and characterize cell proliferation and arrest (using the same methods as in this work). It is thus misleading and incorrect that they "provide a first glimpse of the overall organization of cell proliferation and arrest". This first glimpse was presented in their previous work and here they extend it by redoing their analysis on perturbed cells to visualize the effect of different stresses.

3. I asked whether one can interpret the coordinates in the lower dimensional representation using PHATE, mentioning that this is easy to do in simpler methods, such as PCA. The answer was that "the lower dimensional dimensions of PHATE embeddings are not interpretable in the same way that PCA coordinates are."

My question implied that I knew that these two approaches did not work in the same way. However, are the authors saying that the PHATE coordinates cannot be interpreted in any way (even if it is more complicated and nonlinear vs. the linear combinations of coordinates in a typical PCA)? The (lack of) interpretation of the embeddings in the manuscript is also not helped by the fact that the authors use different projections for different experimental conditions, where only the topography (a cyclical proliferative trajectory and linear arrest trajectory) remain the same.

Reviewer #3:

The authors have adequately addressed my concerns in the revised version of the paper.

Please find attached our revised manuscript, “The molecular architecture of cell cycle arrest”. We have successfully addressed both the reviewers’ and editorial suggestions highlighted in your previous letter.

- In response to the first point raised by Reviewer 2, we would like to highlight the final paragraph of the introduction where we list the novel observations made in this manuscript. We also highlight our novel findings throughout the discussion and describe how these observations add to our understanding of the cell cycle. We take Reviewer 2’s criticisms seriously but are unsure how we can articulate the novelty of this study more clearly than we already have. We gladly welcome your input on this matter.

- We have addressed the reviewer’s second point by changing the language of the last sentence of the abstract. In agreement with the reviewer’s comment that our previous manuscript provided a “first glimpse of the overall organization of cell proliferation and arrest”, we now state that this current work “provides a more comprehensive understanding” of arrested states.

- Regarding the reviewer’s third point, we now include two sentences in the main text that provide intuition for how to interpret the coordinates in PHATE plots. We note that, when multiple stresses were tested simultaneously, the same PHATE coordinate system was used.

- We have also moved a line from the legend of Figure 5B describing how the cell cycle was annotated (which included a reference to data not shown) to the Methods section.

RE: MSB-2022-11087RR, The molecular architecture of cell cycle arrest

Thank you again for sending us your revised manuscript. We have now evaluated your revision. After carefully rereading the manuscript we think that the description of the new findings seems satisfactory and balanced. I think that overall the performed revisions have addressed the previously raised issues. As such, I am pleased to inform you that your paper has been accepted for publication.

EMBO Press Author Checklist

Corresponding Author Name: Jeremy Purvis	
Journal Submitted to: Molecular Systems Biology	
Manuscript Number: MSB-2022-11087	

USEFUL LINKS FOR COMPLETING THIS FORM
The EMBO Journal - Author Guidelines
EMBO Reports - Author Guidelines
Molecular Systems Biology - Author Guidelines
EMBO Molecular Medicine - Author Guidelines

Reporting Checklist for Life Science Articles (updated January
This checklist is adapted from Materials Design Analysis Reporting (MDAR) Checklist for Authors. MDAR establishes a minimum set of requirements in transparent reporting in the life sciences (see Statement of Task: 10.31222/osf.io/9sm4x). Please follow the journal's guidelines in preparing your
Please note that a copy of this checklist will be published alongside your article.

Abridged guidelines for figures
1. Data
The data shown in figures should satisfy the following conditions:
- the data were obtained and processed according to the field's best practice and are presented to reflect the results of the experiments in an accurate and unbiased manner.
- ideally, figure panels should include only measurements that are directly comparable to each other and obtained with the same assay.
- plots include clearly labeled error bars for independent experiments and sample sizes. Unless justified, error bars should not be shown for technical
- if n<5, the individual data points from each experiment should be plotted. Any statistical test employed should be justified.
- Source Data should be included to report the data underlying figures according to the guidelines set out in the authorship guidelines on Data

2. Captions
Each figure caption should contain the following information, for each panel where they are relevant:
- a specification of the experimental system investigated (eg cell line, species name).
- the assay(s) and method(s) used to carry out the reported observations and measurements.
- an explicit mention of the biological and chemical entity(ies) that are being measured.
- an explicit mention of the biological and chemical entity(ies) that are altered/varied/perturbed in a controlled manner.
- the exact sample size (n) for each experimental group/condition, given as a number, not a range;
- a description of the sample collection allowing the reader to understand whether the samples represent technical or biological replicates (including how many animals, litters, cultures, etc.).
- a statement of how many times the experiment shown was independently replicated in the laboratory.
- definitions of statistical methods and measures:
  - common tests, such as t-test (please specify whether paired vs. unpaired), simple χ2 tests, Wilcoxon and Mann-Whitney tests, can be unambiguously identified by name only, but more complex techniques should be described in the methods section;
  - are tests one-sided or two-sided?
  - are there adjustments for multiple comparisons?
  - exact statistical test results, e.g., P values = x but not P values < x;
  - definition of 'center values' as median or average;
  - definition of error bars as s.d. or s.e.m.

Please complete ALL of the questions below.
Select "Not Applicable" only when the requested information is not relevant for your study.

Materials

Newly Created Materials	Information included in the manuscript?	In which section is the information available? (Reagents and Tools Table, Materials and Methods, Figures, Data Availability Section)
New materials and reagents need to be available; do any restrictions apply?	Yes	Materials and Methods

Antibodies	Information included in the manuscript?	In which section is the information available? (Reagents and Tools Table, Materials and Methods, Figures, Data Availability Section)
For **antibodies** provide the following information:<br>- Commercial antibodies: RRID (if possible) or supplier name, catalogue number and or/clone number<br>- Non-commercial: RRID or citation	Yes	Table EV1

DNA and RNA sequences	Information included in the manuscript?	In which section is the information available? (Reagents and Tools Table, Materials and Methods, Figures, Data Availability Section)
**Short novel DNA or RNA including primers, probes**: provide the sequences.	Not Applicable	

Cell materials	Information included in the manuscript?	In which section is the information available? (Reagents and Tools Table, Materials and Methods, Figures, Data Availability Section)
**Cell lines:** Provide species information, strain. Provide accession number in repository **OR** supplier name, catalog number, clone number, and/**OR** RRID.	Yes	Materials and Methods
**Primary cultures:** Provide species, strain, sex of origin, genetic modification status.	Not Applicable	
Report if the cell lines were recently **authenticated** (e.g., by STR profiling) and tested for mycoplasma contamination.	Yes	Materials and Methods

Experimental animals	Information included in the manuscript?	In which section is the information available? (Reagents and Tools Table, Materials and Methods, Figures, Data Availability Section)
**Laboratory animals or Model organisms:** Provide species, strain, sex, age, genetic modification status. Provide accession number in repository **OR** supplier name, catalog number, clone number, **OR** RRID.	Not Applicable	
**Animal observed in or captured from the field:** Provide species, sex, and age where possible**.**	Not Applicable	
Please detail **housing and husbandry conditions**.	Not Applicable	

Plants and microbes	Information included in the manuscript?	In which section is the information available? (Reagents and Tools Table, Materials and Methods, Figures, Data Availability Section)
**Plants:** provide species and strain, ecotype and cultivar where relevant, unique accession number if available, and source (including location for collected wild specimens).	Not Applicable	
**Microbes:** provide species and strain, unique accession number if available, and source.	Not Applicable	

Human research participants	Information included in the manuscript?	In which section is the information available? (Reagents and Tools Table, Materials and Methods, Figures, Data Availability Section)
If collected and within the bounds of privacy constraints report on age, sex and gender or ethnicity for all study participants.	Not Applicable	

Core facilities	Information included in the manuscript?	In which section is the information available? (Reagents and Tools Table, Materials and Methods, Figures, Data Availability Section)
If your work benefited from core facilities, was their service mentioned in the acknowledgments section?	Not Applicable	

Design

**Study protocol**	**Information included in the manuscript?**	**In which section is the information available?** (Reagents and Tools Table, Materials and Methods, Figures, Data Availability Section)
If study protocol has been **pre-registered, provide DOI in the manuscript**. For clinical trials, provide the trial registration number **OR** cite DOI.	Not Applicable	
Report the **clinical trial registration number** (at ClinicalTrials.gov or equivalent), where applicable.	Not Applicable	

**Laboratory protocol**	**Information included in the manuscript?**	**In which section is the information available?** (Reagents and Tools Table, Materials and Methods, Figures, Data Availability Section)
Provide DOI OR other citation details if **external detailed step-by-step protocols** are available.	Yes	Materials and Methods

**Experimental study design and statistics**	**Information included in the manuscript?**	**In which section is the information available?** (Reagents and Tools Table, Materials and Methods, Figures, Data Availability Section)
Include a statement about **sample size** estimate even if no statistical methods were used.	Yes	Figure Legends
Were any steps taken to minimize the effects of subjective bias when allocating animals/samples to treatment (e.g. **randomization procedure**)? If yes, have they been described?	Not Applicable	
Include a statement about **blinding** even if no blinding was done.	Not Applicable	
Describe **inclusion/exclusion criteria** if samples or animals were excluded from the analysis. Were the criteria pre-established?<br><br>If sample or data points were omitted from analysis, report if this was due to attrition or intentional exclusion and provide justification.	Not Applicable	
For every figure, are **statistical tests** justified as appropriate? Do the data meet the assumptions of the tests (e.g., normal distribution)? Describe any methods used to assess it. Is there an estimate of variation within each group of data? Is the variance similar between the groups that are being statistically compared?	Yes	Figure Legends

**Sample definition and in-laboratory replication**	**Information included in the manuscript?**	**In which section is the information available?** (Reagents and Tools Table, Materials and Methods, Figures, Data Availability Section)
In the figure legends: state number of times the experiment was **replicated** in laboratory.	Yes	Figure Legends
In the figure legends: define whether data describe **technical or biological replicates**.	Yes	Figure Legends

Ethics

**Ethics**	**Information included in the manuscript?**	**In which section is the information available?** (Reagents and Tools Table, Materials and Methods, Figures, Data Availability Section)
Studies involving **human participants**: State details of **authority granting ethics approval** (IRB or equivalent committee(s), provide reference number for approval.	Not Applicable	
Studies involving **human participants**: Include a statement confirming that **informed consent** was obtained from all subjects and that the experiments conformed to the principles set out in the WMA Declaration of Helsinki and the Department of Health and Human Services Belmont Report.	Not Applicable	
Studies involving **human participants:** For publication of **patient photos**, include a statement confirming that consent to publish was obtained.	Not Applicable	
Studies involving experimental **animals**: State details of **authority granting ethics approva**l (IRB or equivalent committee(s), provide reference number for approval. Include a statement of compliance with ethical regulations.	Not Applicable	
Studies involving **specimen and field samples:** State if relevant **permits** obtained, provide details of authority approving study; if none were required, explain why.	Not Applicable	

**Dual Use Research of Concern (DURC)**	**Information included in the manuscript?**	**In which section is the information available?** (Reagents and Tools Table, Materials and Methods, Figures, Data Availability Section)
Could your study fall under dual use research restrictions? Please check biosecurity documents and list of **select agents and toxins** (CDC): https://www.selectagents.gov/sat/list.htm	Not Applicable	
If you used a select agent, is the security level of the lab appropriate and reported in the manuscript?	Not Applicable	
If a study is subject to dual use research of concern regulations, is the name of the **authority granting approval and reference number** for the regulatory approval provided in the manuscript?	Not Applicable	

Reporting

The MDAR framework recommends adoption of discipline-specific guidelines, established and endorsed through community initiatives. Journals have their own policy about requiring specific guidelines and recommendations to complement MDAR.

**Adherence to community standards**	**Information included in the manuscript?**	**In which section is the information available?** (Reagents and Tools Table, Materials and Methods, Figures, Data Availability Section)
State if relevant guidelines or checklists (e.g., **ICMJE, MIBBI, ARRIVE, PRISMA**) have been followed or provided.	Not Applicable	
For **tumor marker prognostic studies,** we recommend that you follow the **REMARK** reporting guidelines (see link list at top right). See author guidelines, under 'Reporting Guidelines'. Please confirm you have followed these guidelines.	Not Applicable	
For **phase II and III randomized controlled trials**, please refer to the **CONSORT** flow diagram (see link list at top right) and submit the CONSORT checklist (see link list at top right) with your submission. See author guidelines, under 'Reporting Guidelines'. Please confirm you have submitted this list.	Not Applicable	

Data Availability

**Data availability**	**Information included in the manuscript?**	**In which section is the information available?** (Reagents and Tools Table, Materials and Methods, Figures, Data Availability Section)
Have **primary datasets** been deposited according to the journal's guidelines (see 'Data Deposition' section) and the respective accession numbers provided in the Data Availability Section?	Yes	Data availability
Were **human clinical and genomic datasets** deposited in a public access-controlled repository in accordance to ethical obligations to the patients and to the applicable consent agreement?	Not Applicable	
Are **computational models** that are central and integral to a study available without restrictions in a machine-readable form? Were the relevant accession numbers or links provided?	Not Applicable	
If publicly available data were reused, provide the respective **data citations in the reference list**.	Not Applicable	
